# ESNOQ, Proteomic Quantification of Endogenous *S*-Nitrosation

**DOI:** 10.1371/journal.pone.0010015

**Published:** 2010-04-02

**Authors:** Xixi Zhou, Peiwei Han, Jiangmei Li, Xu Zhang, Bo Huang, Hong-Qiang Ruan, Chang Chen

**Affiliations:** 1 National Laboratory of Biomacromolecules, Institute of Biophysics, Chinese Academy of Sciences, Beijing, China; 2 Key Laboratory of Systems Biology, Institute of Biochemistry and Cell Biology, Shanghai Institutes for Biological Sciences, Chinese Academy of Sciences, Shanghai, China; The Research Institute for Children, United States of America

## Abstract

*S*-nitrosation is a post-translational protein modification and is one of the most important mechanisms of NO signaling. Endogenous *S*-nitrosothiol (SNO) quantification is a challenge for detailed functional studies. Here we developed an ESNOQ (Endogenous SNO Quantification) method which combines the stable isotope labeling by amino acids in cell culture (SILAC) technique with the detergent-free biotin-switch assay and LC-MS/MS. After confirming the accuracy of quantification in this method, we obtained an endogenous *S*-nitrosation proteome for LPS/IFN-γ induced RAW264.7 cells. 27 *S*-nitrosated protein targets were confirmed and using our method we were able to obtain quantitative information on the level of *S*-nitrosation on each modified Cys. With this quantitative information, over 15 more *S*-nitrosated targets were identified than in previous studies. Based on the quantification results, we found that the *S*-nitrosation levels of different cysteines varied within one protein, providing direct evidence for differences in the sensitivity of cysteine residues to reactive nitrosative stress and that *S*-nitrosation is a site-specific modification. Gene ontology clustering shows that *S*-nitrosation targets in the LPS/IFN-γ induced RAW264.7 cell model were functionally enriched in protein translation and glycolysis, suggesting that *S-*nitrosation may function by regulating multiple pathways. The ESNOQ method described here thus provides a solution for quantification of multiple endogenous *S*-nitrosation events, and makes it possible to elucidate the network of relationships between endogenous *S*-nitrosation targets involved in different cellular processes.

## Introduction

*S*-nitrosation (commonly referred to as *S-*nitrosylation) has emerged as an important post-translational modification regulating protein functions, and is believed to be ubiquitously involved in cell signaling [1]. Although there are some methods, such as biotin-switch and SNOSID, for studying *S*-nitrosation [2], [3], a high-throughput method for quantifying the level of *S*-nitrosation has not yet been developed. At the present time, there are several limitations associated with current methods for *S*-nitrosation, including: (I) Elimination of false negatives during identification of *S*-nitrosation targets. The criterion for identifying positive *S*-nitrosation targets in the biotin-switch assay is a significant increase in signal relative to the negative control. However, if an *S*-nitrosated target protein is identified in both the sample and the negative control, it must be eliminated from the candidate list without obtaining further information on relative quantification as it is technically difficult to confirm whether there is a significant increase in sample signal relative to the negative control. (II) In endogenous studies the above problem is even more serious because of the low abundance of endogenous *S*-nitrosated proteins. It is very hard to determine *S*-nitrosation of sample proteins because of the background present in the control. (III) In studies of the dynamics of *S*-nitrosation it is not sufficient just to know which protein is *S*-nitrosated; rather it is necessary to determine quantitative changes in *S*-nitrosation during biological processes. Quantitative information, which reflects the degree of change in *S*-nitrosated proteins over time, is essential for further study on the functions of *S*-nitrosated proteins. (IV) In addition, *S*-nitrosation usually occurs on multiple targets *in vivo*. Therefore, to study the role of *S*-nitrosation in a cellular process, multiple targets should be considered simultaneously as an integrated network. Taking these factors into account it is clear that there is an urgent need for a quantitative high-throughput method for studying *S*-nitrosation.

Biotin-switch assay is a method for indirect *S*-nitrosation detection. Free thiols were blocked by methylmethanethiosulfonate (MMTS). Then S-nitrosothiols were reduced by ascorbate and labeled by biotin-HPDP. *S*-nitrosated cysteines are converted to biotinylated cysteines accordingly. Stable isotope labeling by amino acids in cell culture (SILAC) [4] is a metabolic labeling strategy that relies on the incorporation of amino acids containing substituted stable isotopes into proteins in living cells for relative quantification by mass spectrometry. SILAC has been widely used for studying dynamics of protein abundance [5] and post-translational modifications [6], such as methylation [7], [8] and phosphorylation [9], [10]. In this work we developed a method of protein *S*-nitrosation quantification termed ESNOQ (Endogenous *S*-nitrosothiol (SNO) Quantification) based on combining the detergent-free biotin-switch method [11], SILAC and LC-MS/MS. Using this method, we have measured the endogenous *S*-nitrosation proteome in LPS/IFN-γ induced RAW264.7 cells [12], [13].

## Results

### Development of the ESNOQ method

**Figure 1:**
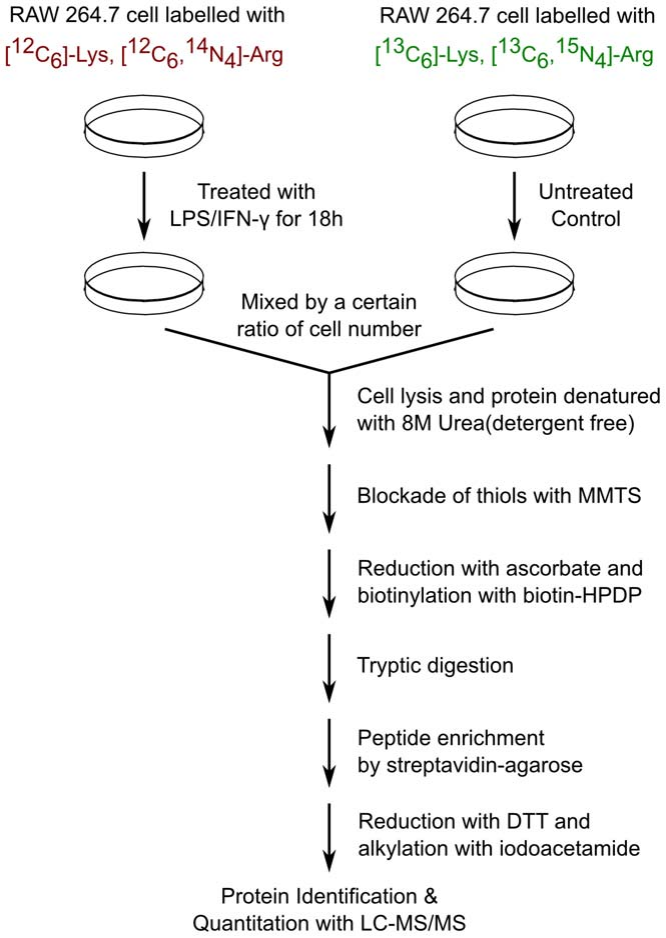
Schematic overview of the ESNOQ strategy. Light or heavy isotope labeled amino acids (Lys, +6 Da; Arg, +10 Da) were incorporated into RAW264.7 cells. Light isotope-labeled cells were then induced by LPS/IFN-γ (treatment group) and mixed with the heavy isotope-labeled cells (control group). After cell lysis, a detergent-free biotin-switch assay, including blockage, reduction and biotinylation steps, was performed. Tryptic digestion was performed before purification to preserve modified site information. After alkylation, the purified peptides were analyzed by LC-MS/MS.

The ESNOQ strategy is shown schematically in Fig. 1. In order to quantify endogenous *S-*nitrosated cysteines via a mass spectrometry approach, we introduced a mass difference between sample proteins and the control. First, light isotopes, [^12^C_6_]-Lys, [^12^C_6_, ^14^N_4_]-Arg, and heavy isotopes, [^13^C_6_]-Lys (+6 Da), [^13^C_6_, ^15^N_4_]-Arg (+10 Da), were incorporated separately into RAW264.7 cells. This was termed “metabolic labeling”. Cells labeled with light isotopes were then treated with LPS/IFN-γ for 18 h, while cells labeled with heavy isotopes were used as controls. The reason why light isotope-labeled cells were used as the treatment group is to avoid excess utilization of Arg by iNOS during nitric oxide synthesis. Treated cells were then mixed with the control group at a ratio of 1∶2 on the basis of cell number. We used the ratio 1∶2 instead of 1∶1 to increase the level of the signal in the control group in order to increase the likelihood of detecting S-nitrosation events in the control and thus obtain quantifiable results. The mixed cells were then lysed and proteins were extracted in detergent-free conditions in order to optimize LC-MS/MS analysis [11]. After biotin-switch, tryptic digestion and purification, the peptides which now contain both quantitative and site-specific information were then identified by mass spectrometry.

Before the ESNOQ assay, we confirmed that nitric oxide synthase was significantly induced in RAW264.7 cells by LPS/IFN-γ [14] using Western-blotting and proved that isotope labeling had no influence on cell growth and properties, in agreement with previous reports [15]. The incorporation ratio of heavy isotopes in every cell generation was also monitored by LC-MS/MS (see Fig. S1 in Supporting Information). In the 6^th^ generation, the signal of light isotope-labeled peptides was below the level of detection for MS, indicating the full incorporation of heavy isotope labeled amino acids.

### Validation of the ESNOQ method

**Figure 2:**
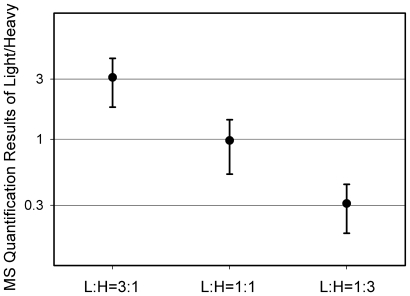
Accuracy of the ESNOQ method. MS results obtained for quantification of *S*-nitrosation at different ratios of light/heavy-labeled cells are shown. Three groups: L:H 3∶1, 1∶1 and 1∶3, were quantified using the ESNOQ method. The solid circle represents the mean of quantification result of each group. Bottom and top bars indicate the standard deviation (SD) of *S*-nitrosation quantification values of each group.

In the ESNOQ assay it is important to preserve accurate *S-*nitrosation quantitative information after the biotin-switch and purification steps. To test whether our method does preserve accuracy, the same concentration of proteins extracted from RAW264.7 cells labeled with light and heavy isotopes were both treated with 1 mM S-nitrosoglutathione (GSNO) in the dark for 1 h. Then the light and heavy-isotope labeled samples were mixed at ratios of 3∶1, 1∶1 and 1∶3. After ESNOQ analysis and database searching, data were validated by Peptideprophet [16]: peptides were accepted when they satisfied all of the following conditions: Peptideprophet *p*>0.8; peptides in both the heavy and light forms had the same elution time; more than four continuous *b* ions or *y* ions were matched. All data were manually checked. Fig. 2 shows the mean and standard deviation (SD) of each sample. For example, in the L:H 1∶1 group, the solid circle shows the mean of the actual MS quantification results of light/heavy of all peptides in this group, which is expected to be equaled to 1. The standard deviation of this sample is about 0.5. And the standard deviations of the L: H 3∶1 group and the L: H 1∶3 group are about 1 and 0.1, respectively.

### Quantification of Endogenous *S-*nitrosation in RAW264.7 Cells induced by LPS/IFN-γ with the ESNOQ Method

**Figure 3:**
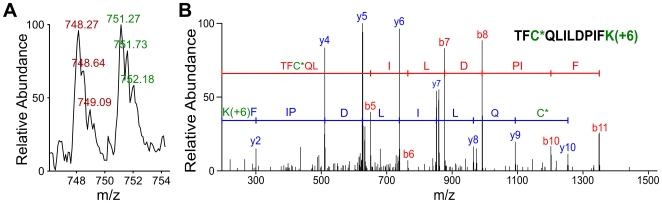
Quantification and identification of *S*-nitrosation targets in RAW264.7 cells induced by LPS/IFN-γ. Peptide TFCQLILDPIFK from Elongation Factor 2 (Swiss-prot: P58252) is shown as an example. (**A**) Peaks of light and heavy precursor ions in the same scan. The light and heavy peaks, and even individual isotope peaks, were clearly separated. The relative abundance of the peaks corresponds to the amount of S-nitrosation. (**B**) Peptide sequence identification by MS^2^. The precursor ion was the heavy isotope labeled peptide, all single charged y ions had a +6 m/z shift, and the cysteine with a +57 m/z shift was matched with carboxyamidomethylation due to alkylation with idoacetamide.

Samples were prepared as shown in Fig. 1 and LC-MS/MS detection was performed followed by database searching, data validation and quantification. SEQUEST™ was used for data searching with the IPI mouse 3.29 database. Peptides were then validated using Peptideprophet: peptides containing cysteine and having a Peptideprophet probability higher than 0.80 were accepted (Due to the limitation of purification efficiency, there were about 20% non-cysteine containing peptides in this study). With this restriction, the sensitivity of peptide identification was above 50% (i.e. more than 50% “true” peptides were identified) and the error rate was 4% (i.e. 4% of the peptides in the results could be errors). ASAPRatio [17] was used to integrate the peak of the peptide in the extracted ion current (XIC) for quantifying the signals. Taking the peptide TFCQLILDPIFK from Elongation factor 2 (Swiss-prot, P58252) as an example, the three left peaks in red in Fig. 3A are the MS^1^ signals of light isotope-labeled peptides. The heavy isotope-labeled peptides in green have a +3 m/z shift because the [^13^C_6_]-Lys is +6 Da heavier in mass compared to [^12^C_6_]-Lys. The ratio calculated by ASAPRatio was 1.0. Thus the quantity of *S-*nitrosation of the Cys in TFCQLILDPIFK from Elongation Factor 2 was 2.0 since the treated cells were mixed with control group cells in a ratio of 1∶2 based on cell number, indicating that the *S*-nitrosation of this cysteine increased two-fold after LPS/IFN-γ treatment. This peptide sequence was identified by database searching using results from MS^2^ as shown in Fig. 3B. The precursor ion was the heavy isotope-labeled peptide, all single charged y ions had a +6 m/z shift, and the cysteine also matched with a carboxyamidomethylation with a +57 m/z shift due to alkylation with iodoacetamide. The peptide sequence and the isotope labeling status (light or heavy) were obtained by database search. Then the m/z of its partner peptide was calculated according to the charge state and isotope labeling status. For the peptide pair in Fig. 3, the bivalent heavy peptide was sequenced from MS2, which showed that it was labeled by K (+6). So the light isotope labeled peptide should have −3 m/z shift. Then the elution profiles of the peptide pair were extracted according to the m/z. The ratio of the light and heavy peptide pair was calculated automatically by ASAPRatio according to the peak areas of the extracted ion current of the peptide pairs. After manually checking each set of data, all together 27 protein targets, including 31 different *S-*nitrosation sites, were identified as shown in Table 1. Over 15 *S*-nitrosated proteins were found here for the first time and six ribosomal proteins were newly identified in LPS/IFN-γ induced RAW264.7 cells. Endogenous *S-*nitrosation increased up to 10-fold in this model. MS and MS^2^ information of all peptides enlisted in Table 1 can be found in Supplemental Information (Fig. S2). We have also blasted all the peptides listed in Table 1 and found no peptides were shared by different proteins. We would like to point out that there are two cysteine residues in the peptide IVSNASCTTNCLAPLAK of GAPDH, which the *S*-nitrosation of Cys increased 6.3 times. We are still not able to conclude that which Cys on this peptide was the S-nitrosation site or both are. One way to resolve this issue is to use another digestion method that can cut the peptide between these two Cys residues, and combine with a relevant isotope-labeling strategy.

**Table 1:** *S*-nitrosated proteins in RAW264.7 cells induced by LPS/IFN-γ.

Swiss-prot	Protein Name	Peptide	Ratio	Error of Ratio	Peptideprophet *p*
**Glycolysis**					
P16858	Glyceraldehyde 3-phosphate dehydrogenase	IVSNASCTTNCLAPLAK	6.30	0.28	0.988
		VPTPNVSVVDLTCR	3.08	0.16	1.000
P52480	Pyruvate kinase isozymes M1/M2	CCSGAIIVLTK	6.92	0.52	1.000
		NTGIICTIGPASR	2.66	0.18	1.000
**Translation**					
P62281	40S ribosomal protein S11	CPFTGNVSIR	2.64	0.22	1.000
P27659	60S ribosomal protein L3	VACIGAWHPAR	2.22	0.08	0.986
Q9D8N0	Elongation factor 1-gamma	WFLTCINQPQFR	2.04	0.24	1.000
P58252	Elongation factor 2	TFCQLILDPIFK	2.00	0.14	1.000
		DLEEDHACIPIK	1.30	0.12	0.992
		STLTDSLVCK	1.54	0.06	0.965
P62908	40S ribosomal protein S3	GLCAIAQAESLR	1.80	0.14	1.000
P62911	60S ribosomal protein L32	SYCAEIAHNVSSK	1.70	0.18	1.000
P62242	40S ribosomal protein S8	NCIVLIDSTPYR	15.60	0.40	0.868
P97461	40S ribosomal protein S5	VNQAIWLLCTGAR	1.80	0.08	1.000
**Biosynthetic processes**					
O35215	D-dopachrome decarboxylase	STEPCAHLLVSSIGVVGTAEQNR	2.40	0.20	1.000
Q61753	D-3-phosphoglycerate dehydrogenase	NAGTCLSPAVIVGLLR	1.90	0.14	1.000
P05202	Aspartate aminotransferase	TCGFDFSGALEDISK	1.82	0.16	1.000
Q920E5	Farnesyl pyrophosphate synthetase	CSWLVVQCLLR	1.22	0.08	1.000
**Proteolysis**					
P10605	Cathepsin B	EQWSNCPTIGQIR	3.32	0.24	0.999
O70370	Cathepsin S	SGVYDDPSCTGNVNHGVLVVGYGTLDGK	1.34	0.16	1.000
**Other Processes**					
Q61699	Heat shock protein 105 kDa	GCALQCAILSPAFK	2.84	0.14	1.000
P35700	Peroxiredoxin-1	HGEVCPAGWKPGSDTIKPDVNK	1.50	0.12	1.000
Q60854	Serpin B6	TCDLLASFK	2.28	0.08	0.993
Q62159	Rho-related GTP-binding protein RhoC	TCLLIVFSK	1.98	0.14	0.997
P40142	Transketolase	TVPFCSTFAAFFTR	1.96	0.10	0.995
Q02053	Ubiquitin-like modifier-activating enzyme 1 X	YFLVGAGAIGCELLK	1.90	0.20	1.000
Q93092	Transaldolase	ALAGCDFLTISPK	1.72	0.12	0.996
Q9R0P3	S-formylglutathione hydrolase	CPALYWLSGLTCTEQNFISK	1.74	0.08	1.000
P99029	Peroxiredoxin-5	GVLFGVPGAFTPGCSK	1.50	0.10	0.997
O08807	Peroxiredoxin-4	HGEVCPAGWKPGSETIIPDPAGK	1.26	0.08	0.979
P05213	Tubulin alpha-1B chain	SIQFVDWCPTGFK	1.20	0.12	1.000

In addition, we also quantified *S*-nitrosation of 10 µM GSNO treated RAW264.7 cells with the ESNOQ method. The results can be found in Supplemental Information (Table S1). About 90 S-nitrosation proteins were quantified and the S-nitrosation level increased with the ratios from 1.5 to about 8.0. We found that some S-nitrosated proteins such as pyruvate kinase, elongation factor and ribosomal proteins were also S-nitrosated targets in the LPS/IFN treated RAW264.7 cells, and the S-nitrosation sites are the same. These results proved the reliability of the ESNOQ method.

### Bioinformatics analysis of the *S*-nitrosated proteins

**Figure 4:**
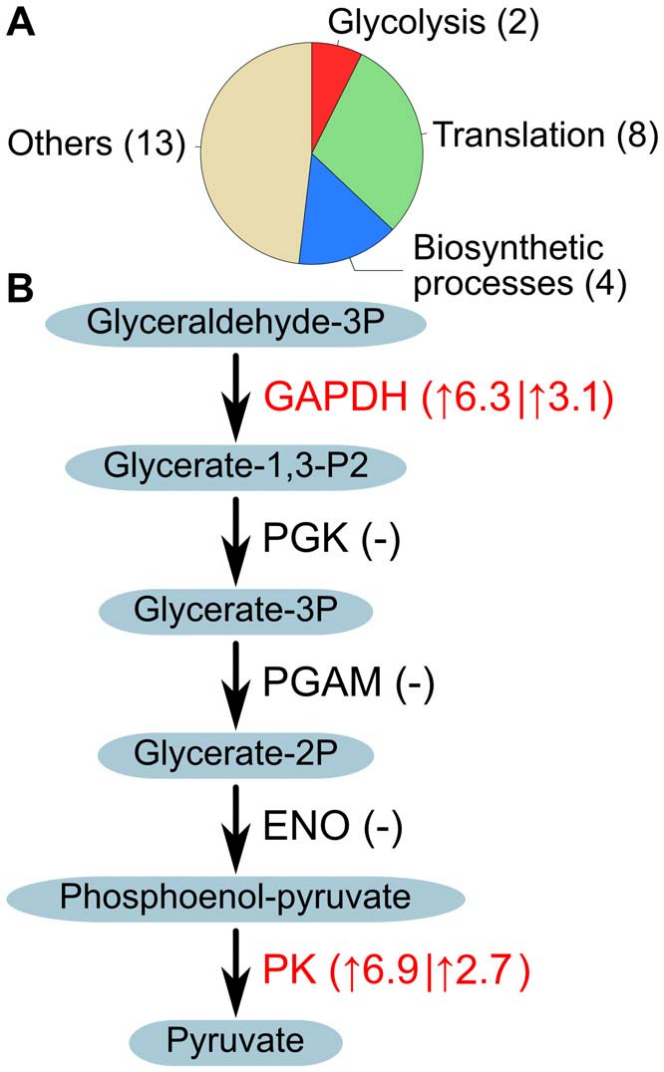
Bioinformatics analysis of *S*-nitrosation targets in RAW264.7 cells induced by LPS/IFN-γ. (**A**) Breakdown of *S*-nitrosated proteins by Gene Ontology category. (**B**) The glycolysis pathway from glyceraldehyde-3P to pyruvate. Two enzymes are *S*-nitrosation targets (in red) and the quantitative levels of *S*-nitrosation are labeled.

The *S-*nitrosated proteins were queried in the Gene Ontology Database (http://www.ebi.ac.uk/ego/) and clustered according to biological processes. As shown in Fig. 4A, the S-nitrosated targets glyceraldehyde 3-phosphate dehydrogenase (GAPDH) and pyruvate kinase (PK) are involved in glycolysis. Four proteins, for example aspartate aminotransferase, were involved in biosynthetic processes. Six ribosomal proteins and two elongation factors were involved in translation. Consistent with the above results, KEGG pathway analysis [18] also revealed two notable *S*-nitrosation-rich pathways: glycolysis and protein translation. The glycolysis pathway, from glyceraldehyde-3P to pyruvate, involves five enzymes, two of which are *S*-nitrosated (in red) (Fig. 4B). Of the 27 *S*-nitrosated proteins detected in this study, GAPDH and PK had high levels of *S*-nitrosation variation. 8 proteins were enriched in the protein translation process.

## Discussion

In this work, we have developed the ESNOQ method and applied it to obtain an endogenous *S*-nitrosation proteome in the LPS/IFN-γ-induced RAW264.7 cell model. Our results have shown that this method is a powerful tool for effectively and accurately quantifying endogenous *S*-nitrosation with specific modified site information. Using ESNOQ, many of the limitations frequently encountered in studies of *S*-nitrosation can be solved: 1) The quantitative information obtained allows identification of *S*-nitrosation protein targets that would otherwise be excluded as false negatives. 2) In endogenous studies, the low abundance of endogenous *S*-nitrosated proteins can be distinguished from the control. 3) More *S*-nitrosated protein targets can be identified with ESNOQ: here we identified 41 different endogenous *S*-nitrosation targets. By way of comparison, only four endogenous *S*-nitrosated protein targets were identified in a similar study using SNOSID [2]. 4) Multiple *S*-nitrosated targets in a given cellular process can be considered simultaneously with this high-throughput method. In brief, *S*-nitrosation signaling mechanisms and pathways can be studied systematically, quantitatively, and site-specifically with this method.

Results from this study are consistent with previous reports; glyceraldehyde-3-phosphate dehydrogenase, pyruvate kinase, and elongation factors 1 and 2 were identified here as *S*-nitrosation targets [13], and the modified sites identified here in glyceraldehyde-3-phosphate dehydrogenase and D-3-phosphoglycerate dehydrogenase were the same as those previously reported [2]. This indicates that the ESNOQ method is reliable. Over 15 of the new *S*-nitrosation targets detected using our method in LPS/IFN-γ treated RAW264.7 cells have not previously been reported [13]. The quantification results showed that the level of *S*-nitrosation of newly identified protein targets increased 1.5 to 2.5 times, while it was previously almost impossible to distinguish these endogenous *S*-nitrosation targets without accurate quantification information.

Quantitative information on site-specific *S*-nitrosation is necessary for in depth functional studies. Using the ESNOQ method, 45 different *S*-nitrosation sites on 41 protein targets were identified in LPS/IFN-γ treated RAW264.7 cells. More interesting, we found that the *S*-nitrosation levels of different cysteines varied within one protein, providing direct evidence for different sensitivity of cysteine residues to reactive nitrosative stress (RNS) and suggesting that *S*-nitrosation is a site-specific modification. Taking GAPDH as an example, the *S*-nitrosation of Cys in the peptide IVSNASCTTNCLAPLAK increased 6.3 times, while *S*-nitrosation increased only 3.1 times at another Cys site (VPTPNVSVVDLTCR). It has been reported that a Cys residue is more easily *S*-nitrosated when it has a lower pKa [1]. Here we calculated the pKa's of cysteines within one protein using PROPKA [19] and found that up-regulation of *S*-nitrosation is tightly correlated with cysteine pKa in the same protein target. In pyruvate kinase, the calculated pKa value of C48 is 11.90, and its *S*-nitrosation ratio is 2.7, while the pKa of C422 is 8.62, significantly lower than that of C48, and its *S*-nitrosation ratio is 6.9, much higher than that of C48. It is the same in GAPDH: C246 has a pKa of 9.24 and an *S*-nitrosation ratio of 3.1, while C151 has an exceptionally low pKa of 5.27 and a high *S*-nitrosation ratio of 6.3. That means, if one protein is known to be an S-nitrosation target, the cysteine with the lowest pKa may be the S-nitrosation site.

With the benefits of high-throughput quantification, the landscape of endogenous *S*-nitrosation has been revealed, which is very important for research on signal transduction mechanisms. Gene ontology clustering of biological processes showed that *S*-nitrosation targets were mainly related to translation and cell metabolism, including biosynthetic processes (e.g. Asparatate aminotransferase), glycolysis (e.g. GAPDH) and proteolysis (e.g. Cathepsin B). In the glycolytic process, 2 enzymes in the same pathway have been identified as *S*-nitrosation targets, and their *S*-nitrosation levels were relatively higher than the other targets. In the translation process, 8 proteins, including 6 ribosomal proteins and 2 elongation factors, were identified as *S*-nitrosation targets. These results indicate that *S-*nitrosation may function by regulating multiple pathways.

Recently an iTRAQ-based quantitative method for *S*-nitrosation detection has been reported [20], however, it has not yet been applied to endogenous analysis. The advantage of iTRAQ approach is that it can be widely used for analysis of cell, tissue and animal samples. However, since the labeling strategy on peptide was carried out after multi-steps of sample preparation, which may introduce significant quantification error, the parallel and accuracy of quantification were compromised. Being different from it, our SILAC-based ESNOQ method shows significant advantages in the parallel and accuracy of quantification because treatment and control group cells can be mixed as intact cells and processed together throughout the experimental procedure. Therefore, sample losses at a particular step do not affect the quantitative accuracy. The follow-up steps including blocking, reducing, labeling and LC-MS analysis are all performed on the same sample. Therefore, ESNOQ has high accuracy for quantification of endogenous SNOs. The disadvantage of our method is that it can not be easily used for animal and tissue samples.

The ESNOQ method described here may be used for analyzing *S*-nitrosation profiles in cellular processes such as apoptosis or differentiation. It could also be used for dynamic studies by labeling with a range of different isotopes. Moreover, the ESNOQ method lends itself to the study of *S*-nitrosated modification networks since multiple SNO targets can now be evaluated using the quantitative information obtained. Thus, the ESNOQ method takes us one step closer to revealing the dynamic endogenous roles of *S*-nitrosation.

## Materials and Methods

### Materials

SILAC™ protein identification and quantitation kits were purchased from Invitrogen (Cat. No. MS10030, USA). *S-*nitrosoglutathione (GSNO) was synthesized as described [21]. Methyl methanethiosulfonate (MMTS), biotin-HPDP (HPDP: N-[6-(biotinamido)hexyl]-3′-(2′-pyridyldithio)propionamido), the BCA™ protein assay kit, and the Slide-A-Lyzer dialysis cassette (0.5 ml to 3 ml, 7 kDa molecular-weight cutoff) were from Pierce (Rockford, IL, USA). PlusOne™ urea was from GE Healthcare (Piscataway, NJ, USA). Protease inhibitor cocktail tablets (Complete-Mini, EDTA-free) were from Roche Applied Sciences (Indianapolis, IN, USA). Sequencing-grade modified trypsin (V5111) was from Promega (Madison, WI, USA). Solvents used in LC/MS analysis, including water, formic acid (FA), acetonitrile (ACN) and methanol, were from J. T. Baker Chemicals (Philipsburg, NJ, USA). All other reagents were purchased from Sigma-Aldrich (St. Louis, MO, USA).

### Cell Culture, Isotope Labeling, and Labeling Efficiency

The RAW264.7 cell line was purchased from Peking Union Medical College (Beijing, China). DMEM (included in the SILAC protein identification and quantitation kit) was prepared according to the manufacturer's instructions. Briefly, the light medium contained 10 mM HEPES, 2 mM L-Glutamine, 100 mg/ml [^12^C_6_]-Lys, 100 mg/ml [^12^C_6_,^14^N_4_]-Arg, 10% dialyzed fetal bovine serum and 100 U/ml penicillin and 100 µg/ml streptomycin. The heavy medium had the same composition except that Lys and Arg were substituted by [^13^C_6_]-Lys and [^13^C_6_,^15^N_4_]-Arg. RAW264.7 cells were grown in the light or heavy isotope labeled medium in a humidified atmosphere with 5% CO_2_ in air at 37°C for 6 generations in order to get effective incorporation. Heavy labeled cells were collected and lysed. After SDS-PAGE of the extracted proteins, all bands were cut out and digested in-gel according to a previously published method [22]. Peptides were extracted into ACN and were ready for analysis by LC-MS/MS to determine labeling rates.

### Validation of the Quantification Method

Light and heavy isotope labeled RAW264.7 cells were lysed with 8 M Urea, and then centrifuged at 12000 g for 20 min. Protein concentration, determined with the BCA assay, was adjusted to 1 mg/ml. Light and heavy labeled protein samples were treated with 1 mM GSNO for 1 h in the dark. Then samples were mixed at ratios of 3∶1, 1∶1 and 1∶3. Samples were then analyzed by LC-MS/MS.

### Preparation of Endogenous *S-*nitrosation Samples

All steps were performed in the dark unless noted otherwise. Light isotope labeled RAW264.7 cells were treated with 100 ng/ml LPS and 1 ng/ml IFN-γ for 18 h, then mixed with heavy isotope labeled RAW264.7 cells at a ratio of 1∶2 on the basis of cell number. Cells were lysed with 8 M urea including 20 mM MMTS in the lysis buffer to avoid the trans-nitrosation followed by centrifugation at 12000 g. Protein concentration was determined with a BCA™ protein assay kit and adjusted to 1 mg/ml. The urea-based biotin-switch method was then performed [11]. Digestion was performed before purification in order to preserve modified site information. After purification with streptavidin agarose and alkylation with iodoacetamide, peptide samples diluted in 0.1% FA were ready for LC-MS/MS analysis.

### Mass Spectrometric Analysis

Samples were analyzed on a Thermo LTQ linear trap instrument equipped with a Thermo micro-electrospray source, a Thermo Surveyor pump and an auto sampler (Thermo Electron Corporation, San Jose, CA, USA), and LC analysis was performed with a 0.15 mm×150 mm (RP-C18) column (Column Technology Inc.). Buffer A consisted of 0.1% FA and buffer B consisted of 0.1% FA in 84% ACN. Peptides were eluted with a linear gradient of buffer B; 4% B was first applied, followed by a linear gradient to 50% B in 105 min, and then to 100% B in 9 min, holding at 100% B for an additional 6 min. MS/MS spectra were acquired using a full scan followed by five MS/MS scans on the five most intense precursor ions in data-dependent mode. MS1 data was collected in profile mode and MS2 data was collected in centroid mode.

### Database Search, Data Validation and Quantification

MS/MS spectra were extracted from the raw data files by TurboSEQUEST™ (licensed to Bioworks 3.3) with the following parameters: molecular weight (MW) range, 400–4000; threshold, 0; precursor mass tolerance, 1.4; fragment ion tolerance, 0.5. The raw data were searched using Turbo SEQUEST with the IPI mouse database 3.29. For the strategy for isotope peaks, we used average molecular weight for precursor ion and monoisotopic for fragment ions. Cysteine carboxyamidomethylation (+57) was specified as a static modification and methionine oxidation (+16), Lys (+6), and Arg (+10) labeling were specified as differential modifications. Search results were validated with the Trans-Proteomics Pipeline (ISB, Seattle, WA) using PeptideProphet. Only peptides with a probability >0.80 were accepted; in this case, the error rate was lower than 4%. Quantification of *S*-nitrosation was performed by ASAPRatio, a program in the Trans-Proteomics Pipeline. The default parameters were employed when using ASAPRatio in our data analysis. In addition, two arguments of ASAPRatio were chosen: (1) use fixed scan range for Light and Heavy. (2) Quantitate only the charge state where the collision-induced dissociation (CID) was made. The ratios were obtained through peak area integration. When a peptide was accepted, the m/z of its partner was inferred according to the sequence and charge state, the mass difference of K is 6 and R is 10. ASAPRatio can locate the LC spectrum of this peptide pair, subtract the background noise and integrate the area of each LC peak of light and heavy peptide. The relative quantification result of this charge state was the ratio of peak areas of light and heavy peptides. The ratios of the same peptide in different charge states are averaged and weighted by the corresponding spectrum intensity to obtain t light/heavy ratio and its error. The actual endogenous *S*-nitrosation ratio is the observed ratio ×2 since the treated cells were mixed with the control group at a ratio of 1∶2 on the basis of cell number.

### Bioinformatics Analysis

PROPKA analysis was performed online (http://propka.ki.ku.dk/) with default parameters. GO enrichment and analysis were performed in Cytoscape (http://www.cytoscape.org) with the Bingo plugin [23]. KEGG pathway enrichment analysis was performed with DAVID [24]. Other data conversions and analysis were performed using in-house Perl scripts.

## Supporting Information

Figure S1 Confirmation of LPS and IFN-γ induced RAW264.7 cells. (A) Western blotting assay indicates that the expression of iNOS was significantly induced after LPS/IFN-γ treatment. (B) Cell morphology at generation 4 shows no difference between light and heavy labeled cells. (C) Quantification of the incorporation of heavy isotope-labeled amino acids via XIC by ASAPRatio. The peptide VAPEEHPVLLTEAPLNPK in Gamma-actin like protein was taken as an example.
(3.19 MB TIF)

Figure S2 ASAPRatio quantification and MS/MS analysis of all peptides listed in Table 1.
(3.17 MB DOC)

Table S1 *S*-nitrosation quantification with the ESNOQ approach of GSNO treated RAW264.7 cells. RAW264.7 cells labeled with heavy amino acids were treated with 10 µM GSNO for 1 h. Cells labeled with light amino acids were used as control group with the solvent as control treatment. First, cell lysis was blocked with MMTS to avoid the trans-nitrosation, and then the two groups were mixed into one sample. Purification and MS/MS analysis were the same as described in the manuscript. The ratios in this table represent the quantification results of Heavy/Light, i.e. the increasing of S-nitrosation after GSNO treatment.
(0.20 MB DOC)
