# Supplementary figures and images for: ESNOQ, Proteomic Quantification of Endogenous S-Nitrosation
Source: PLoS One. 2010 Apr 2;5(4):e10015. doi: 10.1371/journal.pone.0010015 (PMC2848867; doi:10.1371/journal.pone.0010015)

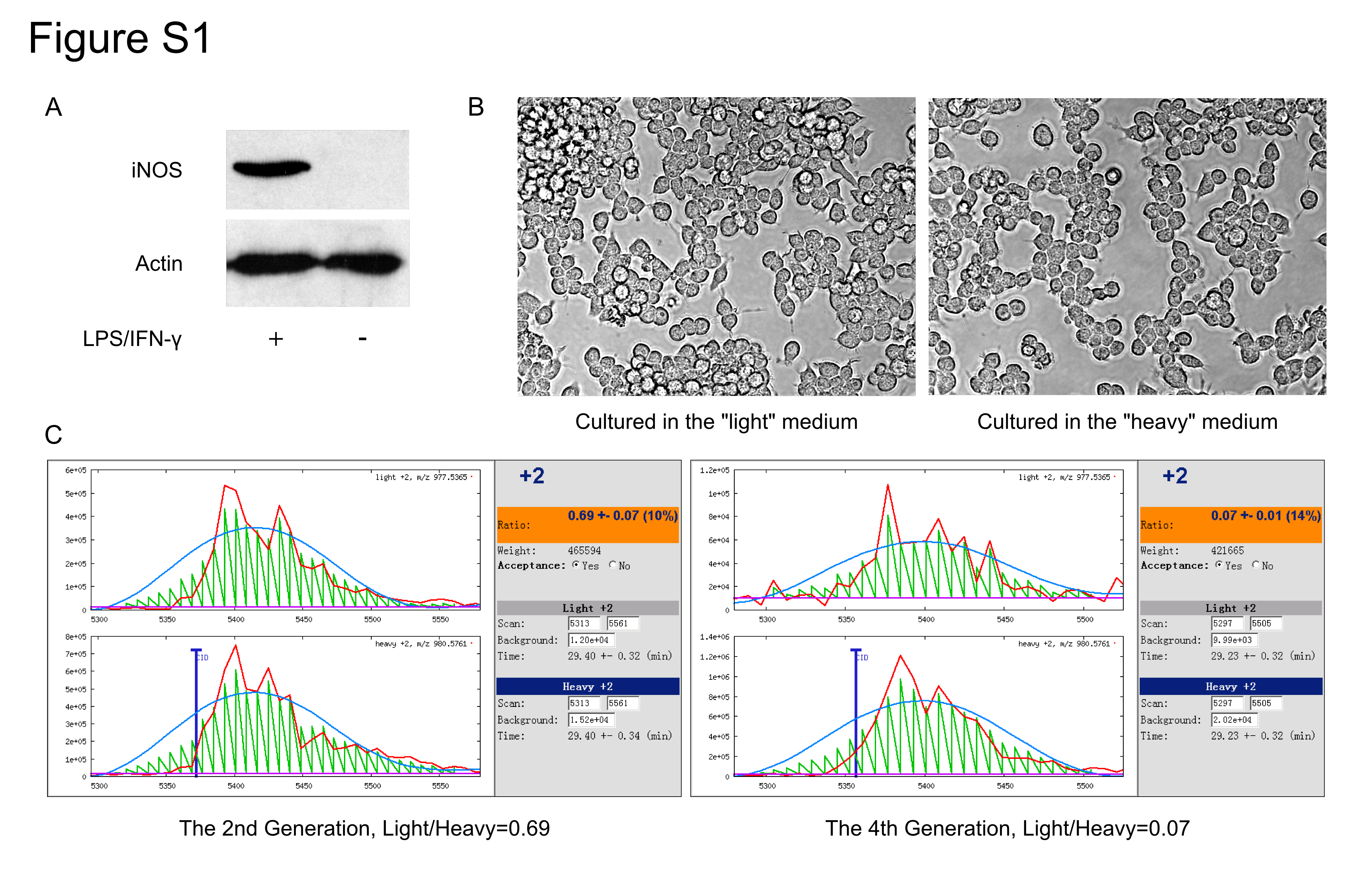

Supplement: Figure S1 — Confirmation of LPS and IFN-γ induced RAW264.7 cells. (A) Western blotting assay indicates that the expression of iNOS was significantly induced after LPS/IFN-γ treatment. (B) Cell morphology at generation 4 shows no difference between light and heavy labeled cells. (C) Quantification of the incorporation of heavy isotope-labeled amino acids via XIC by ASAPRatio. The peptide VAPEEHPVLLTEAPLNPK in Gamma-actin like protein was taken as an example. (3.19 MB TIF) [file pone.0010015.s001.tif]
