# Supplementary material for: ESNOQ, Proteomic Quantification of Endogenous S-Nitrosation
Source: PLoS One. 2010 Apr 2;5(4):e10015. doi: 10.1371/journal.pone.0010015 (PMC2848867; doi:10.1371/journal.pone.0010015)

Figure S2. ASAPRatio quantification and MS/MS analysis of all peptides listed in Table 1.

Glyceraldehyde 3-phosphate dehydrogenase

IVSNASCTTNCLAPLAK


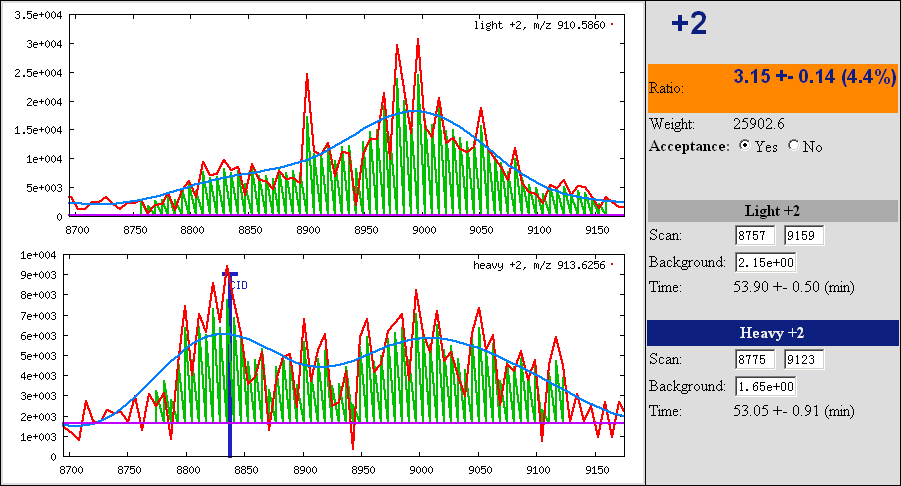


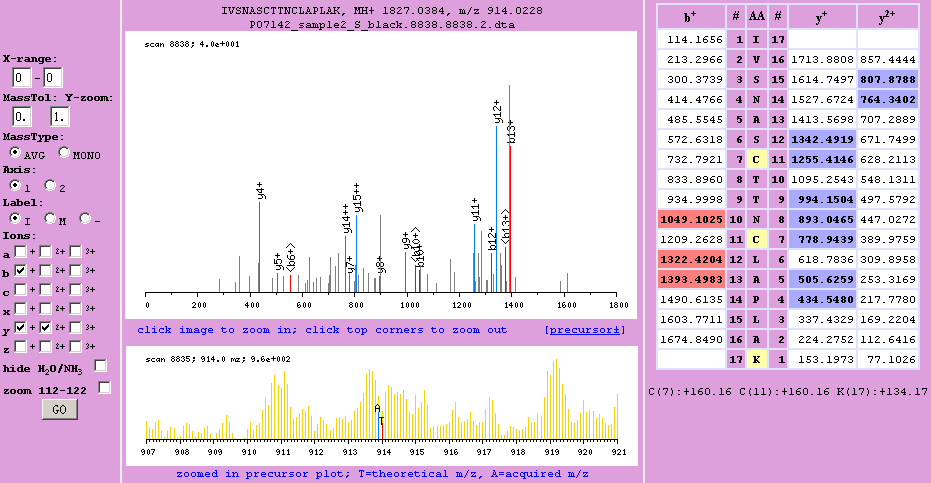


VPTPNVSVVDLTCR


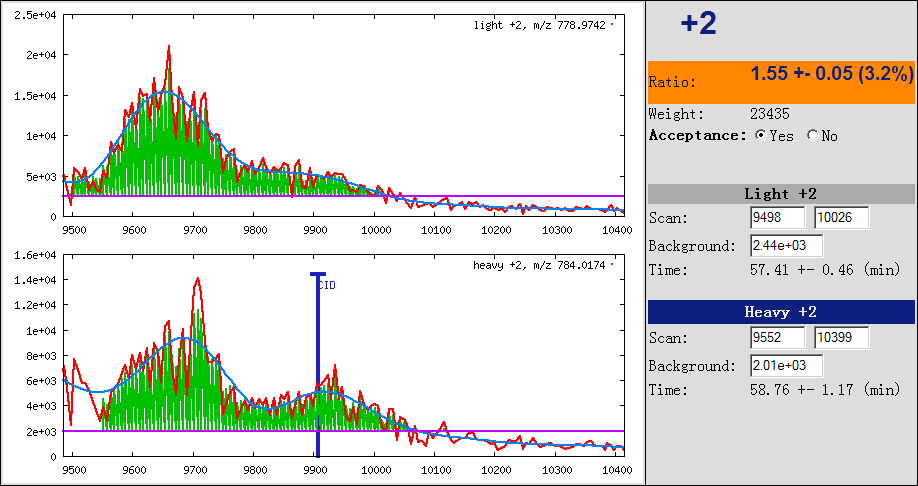

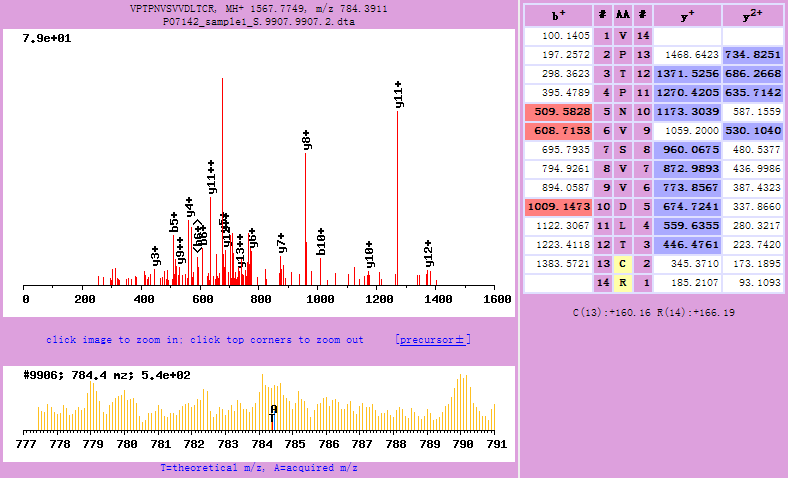


Pyruvate kinase isozymes M1/M2

CCSGAIIVLTK


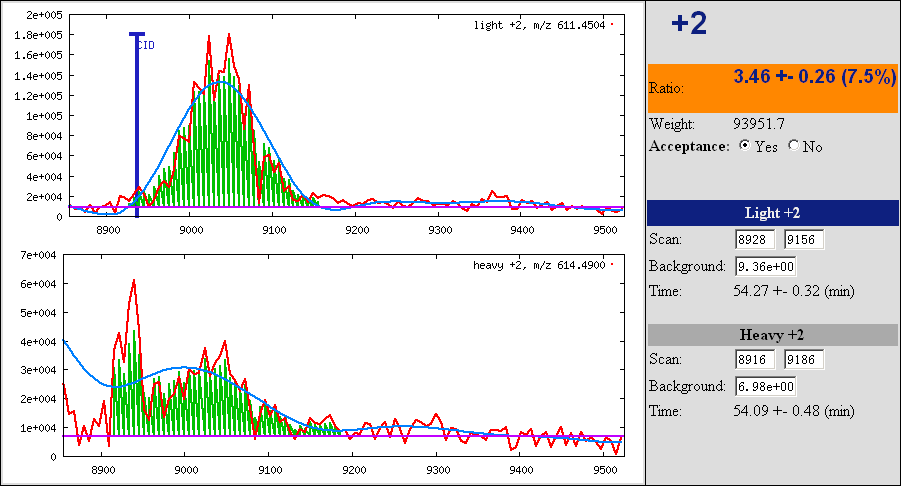


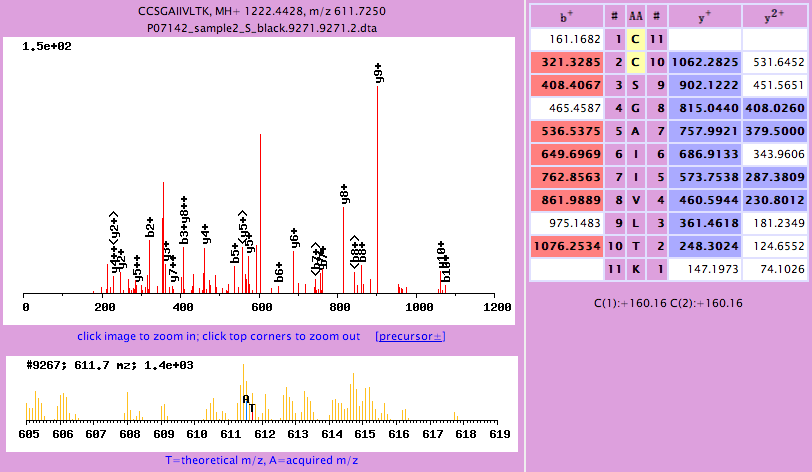


NTGIICTIGPASR


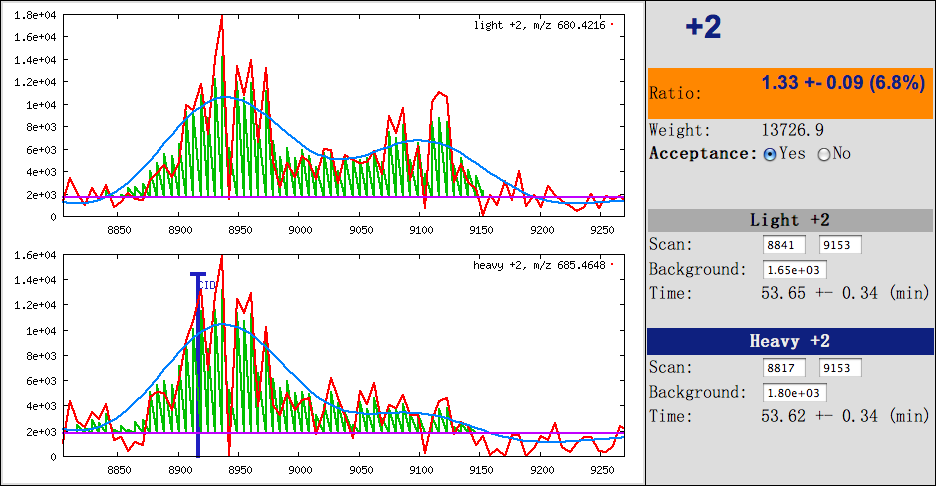

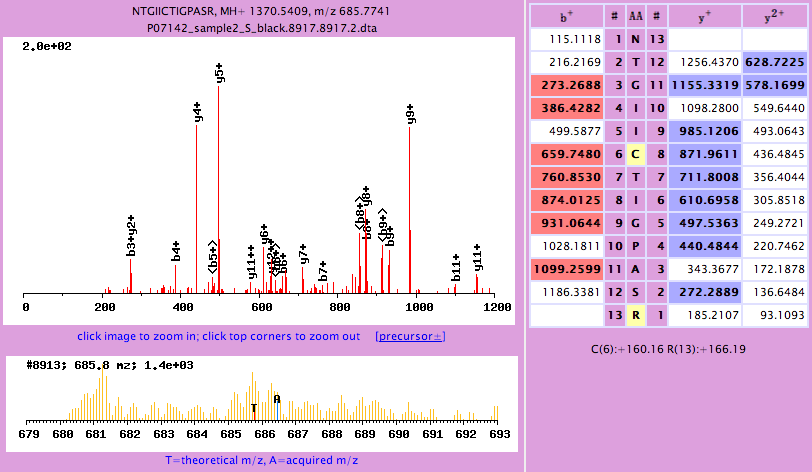


ribosomal protein S11

CPFTGNVSIR


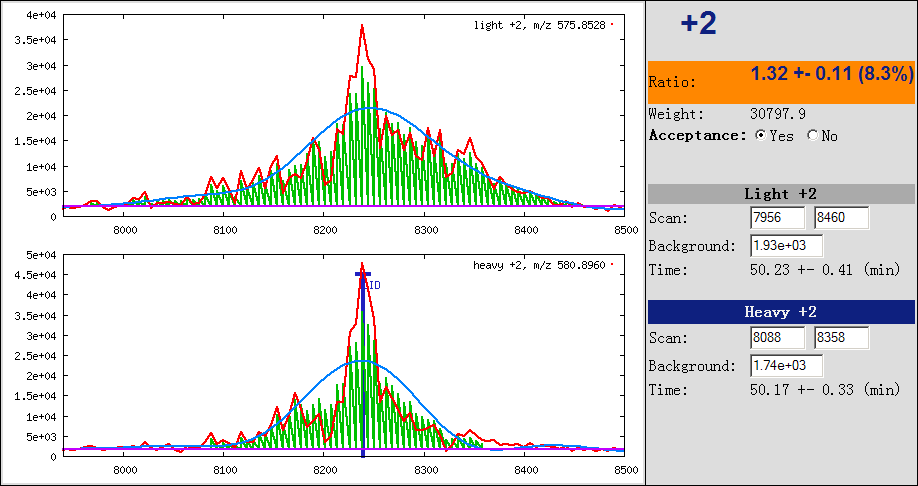

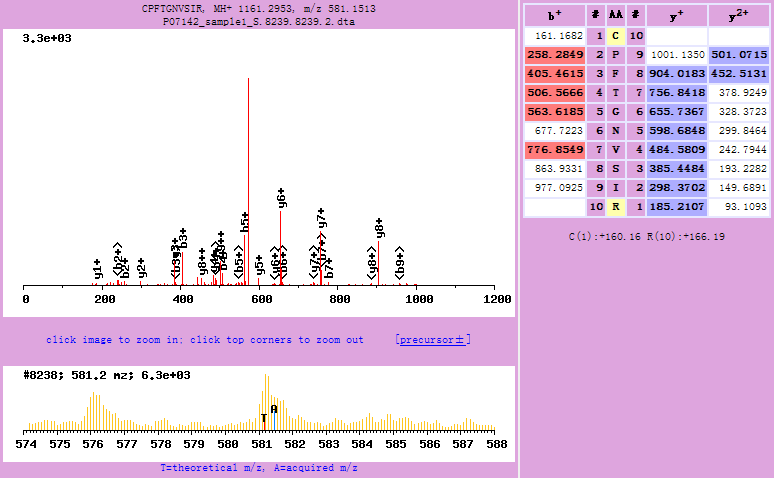


60S ribosomal protein L3

VACIGAWHPAR


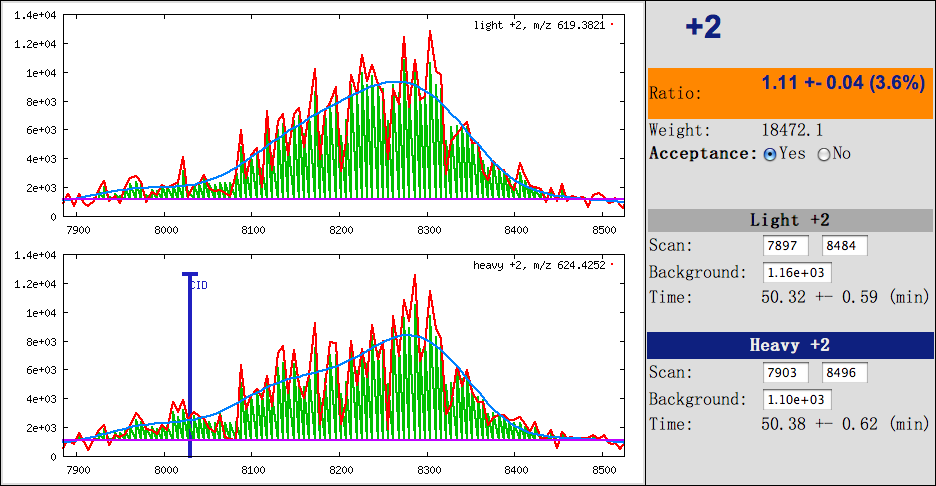

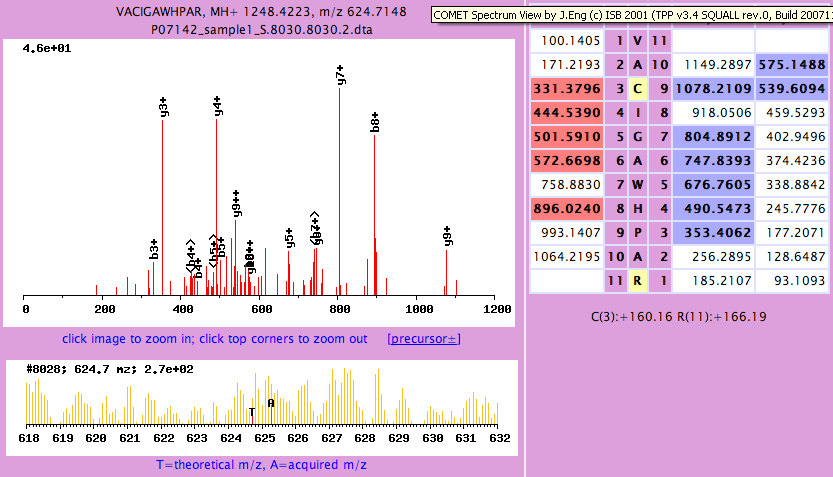


Elongation factor 1-gamma

WFLTCINQPQFR
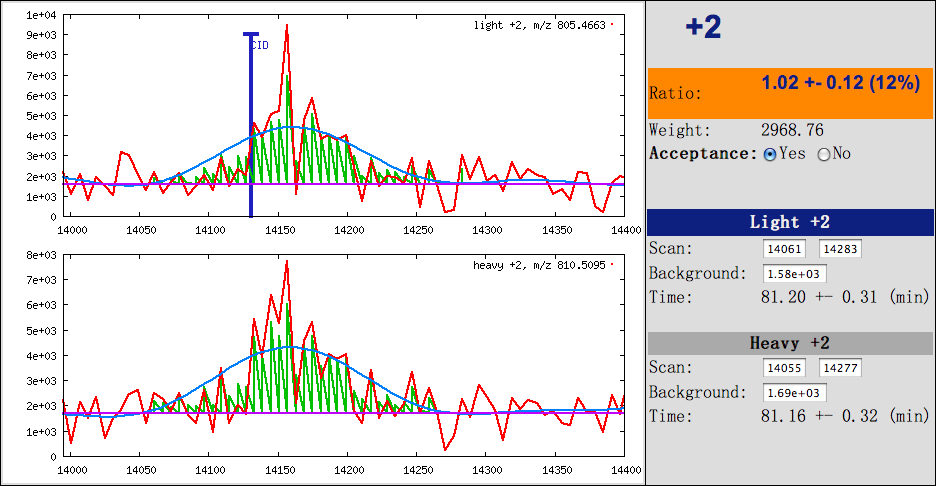

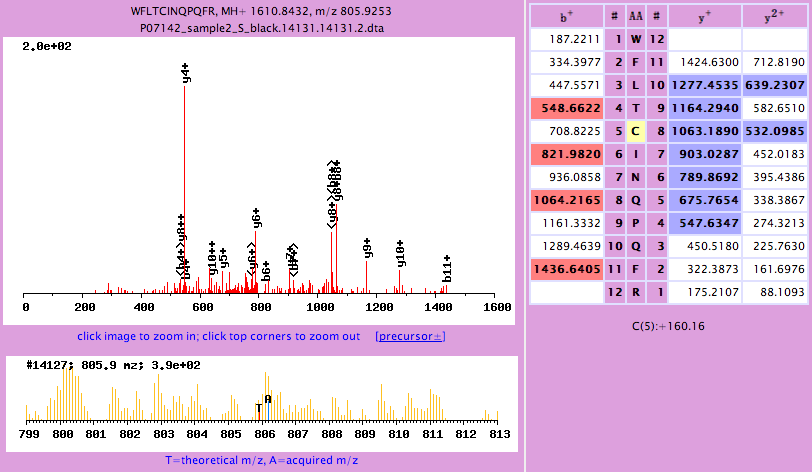


Elongation factor 2

TFCQLILDPIFK


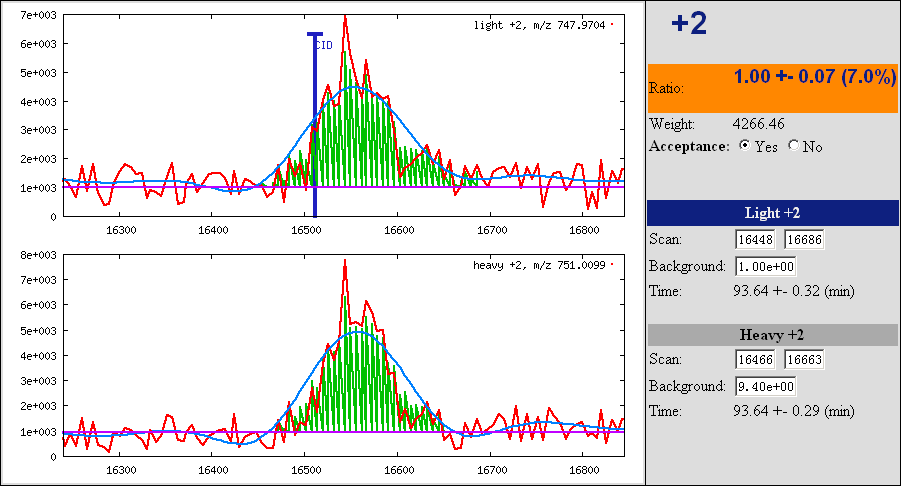

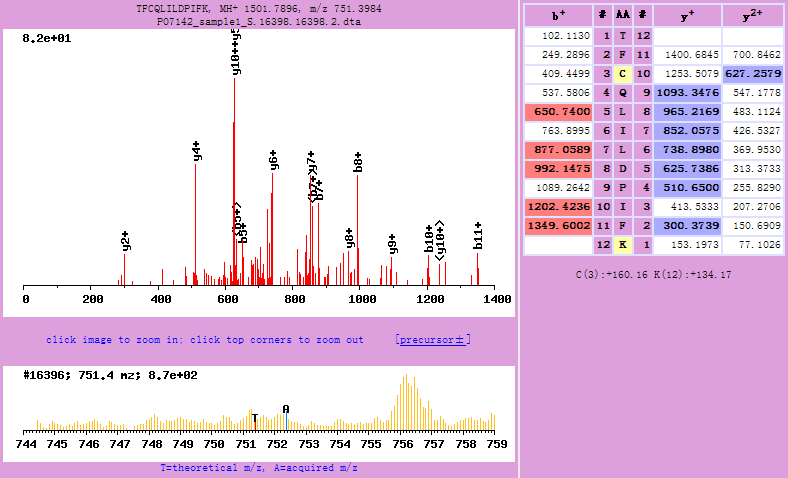


DLEEDHACIPIK


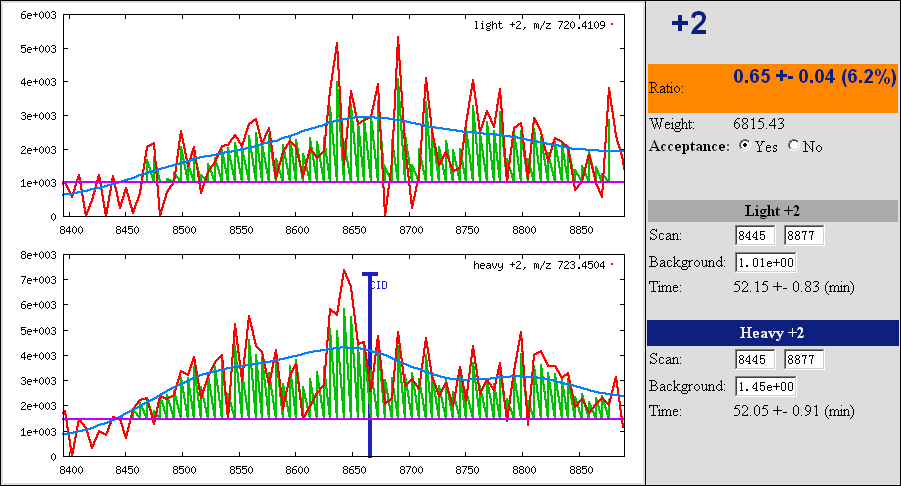


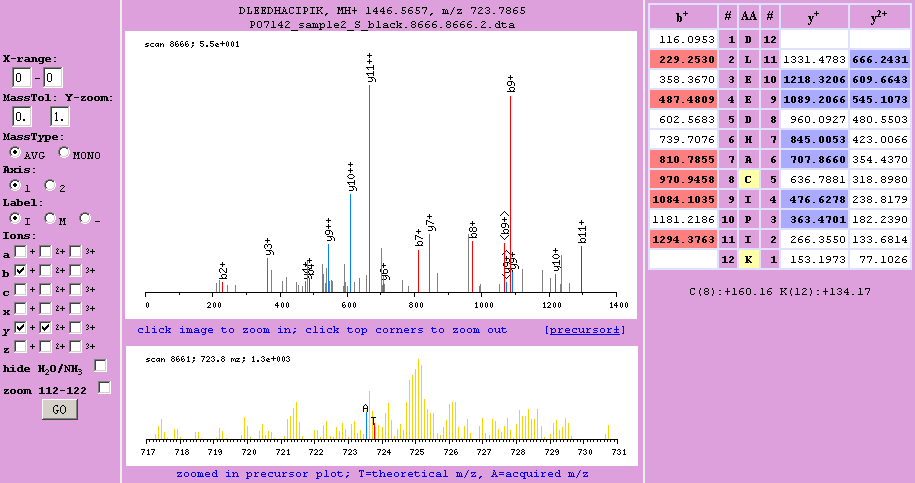


STLTDSLVCK


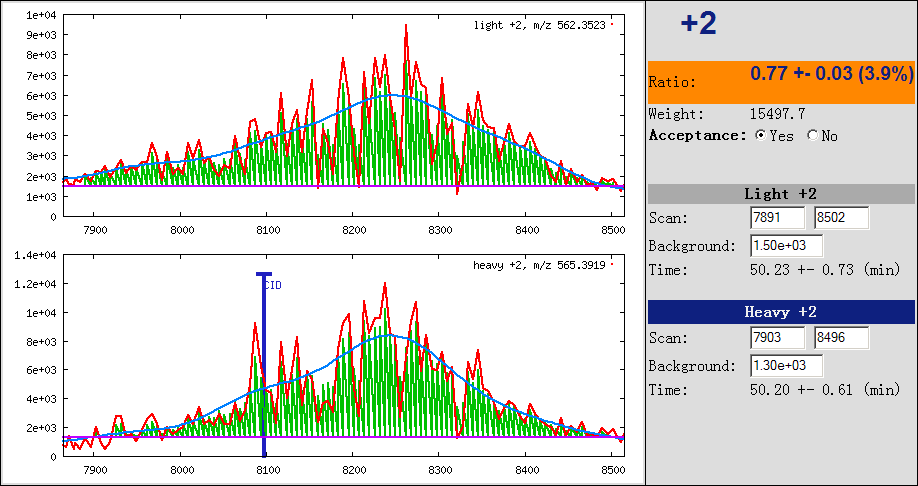

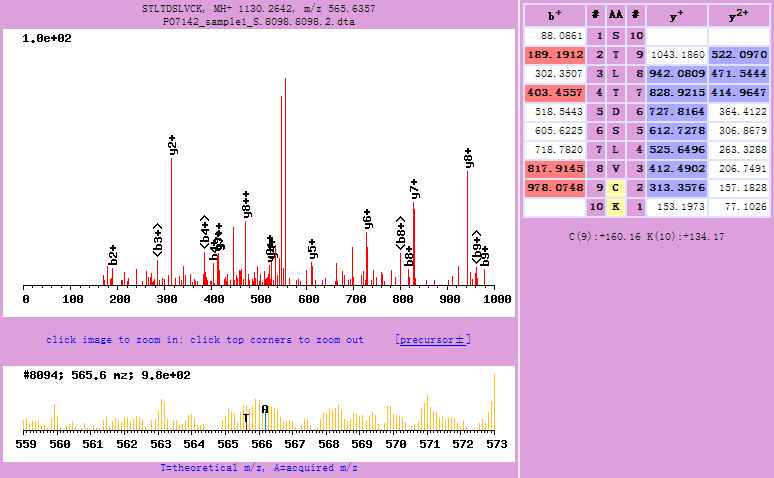


40S ribosomal protein S3

GLCAIAQAESLR


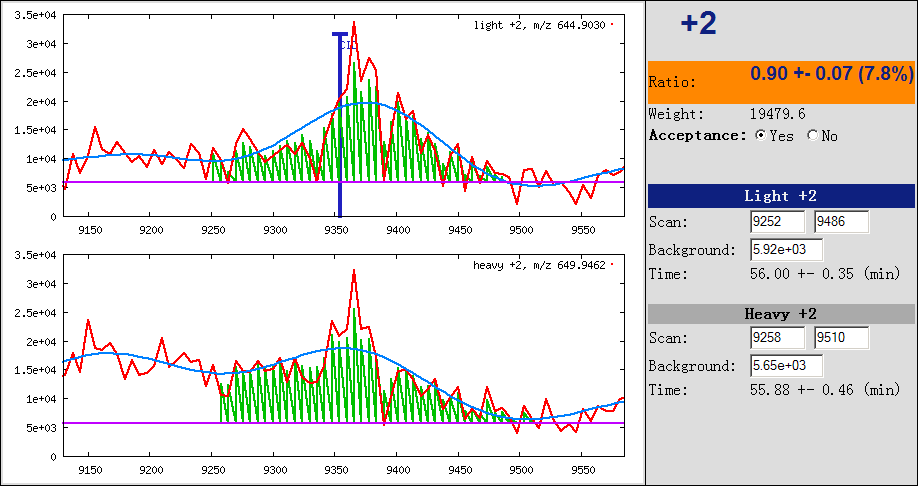

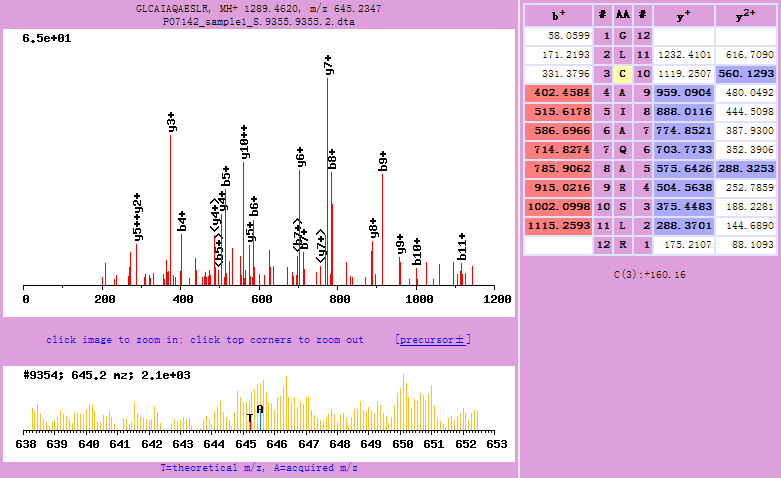


60S ribosomal protein L32

SYCAEIAHNVSSK


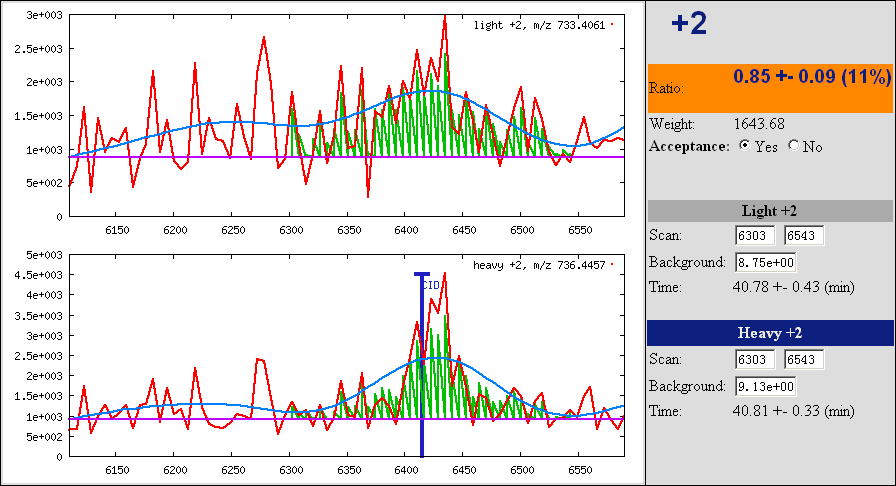


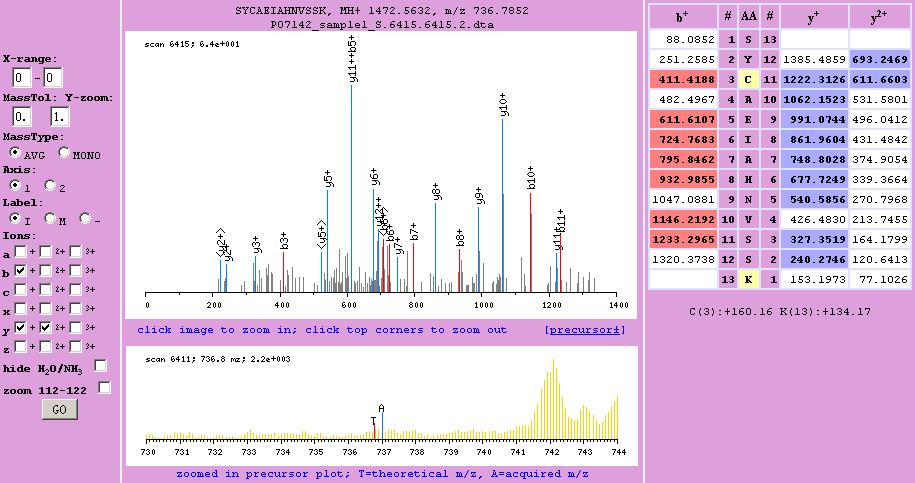


40S ribosomal protein S8

NCIVLIDSTPYR


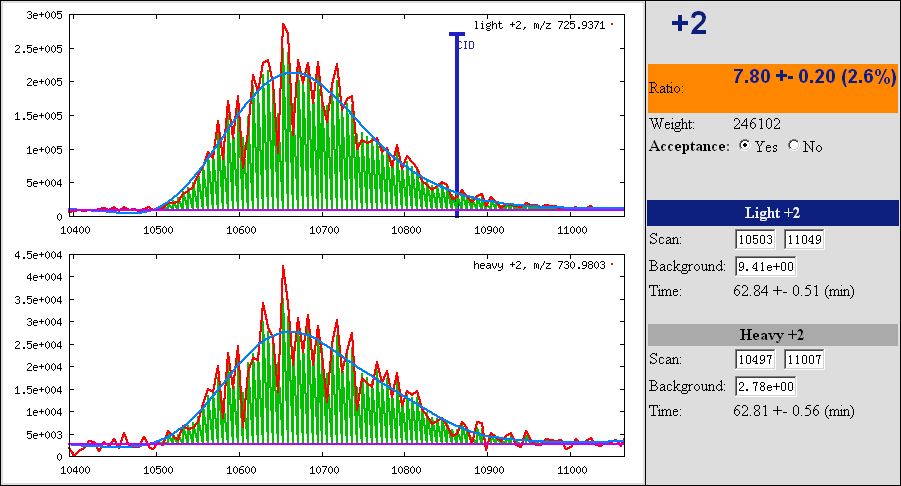


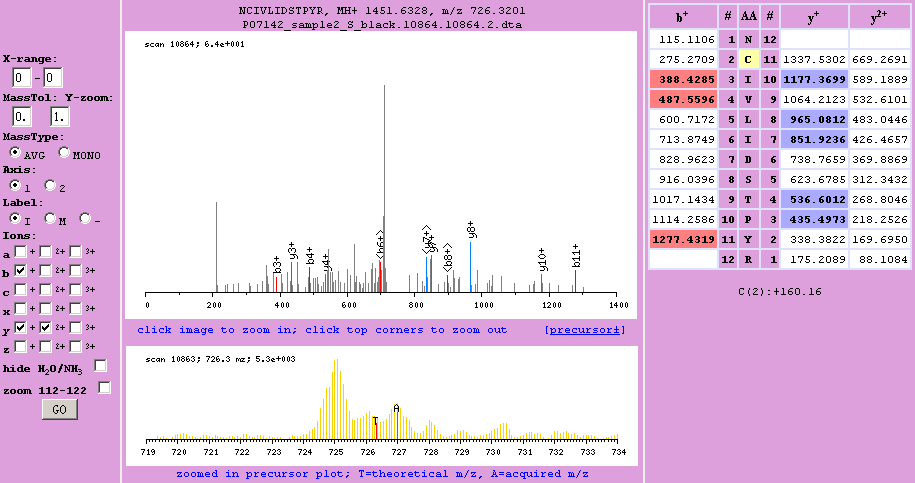


40S ribosomal protein S5

VNQAIWLLCTGAR
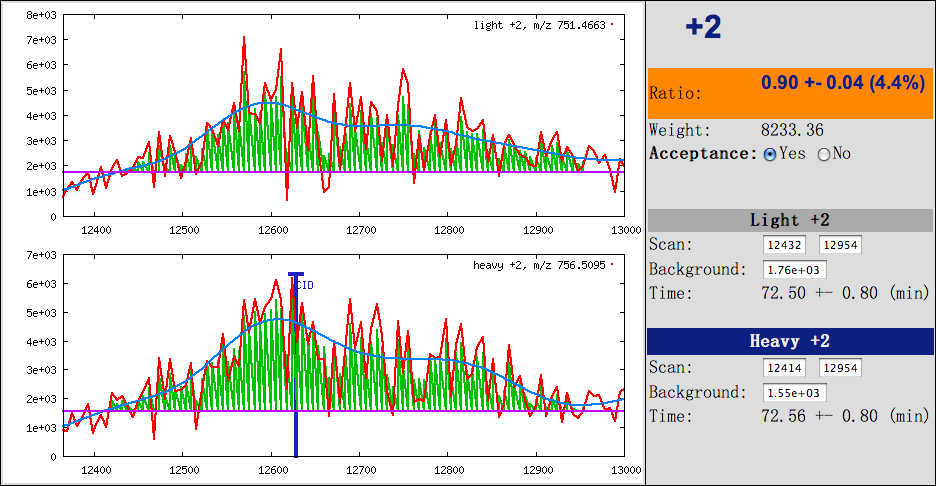

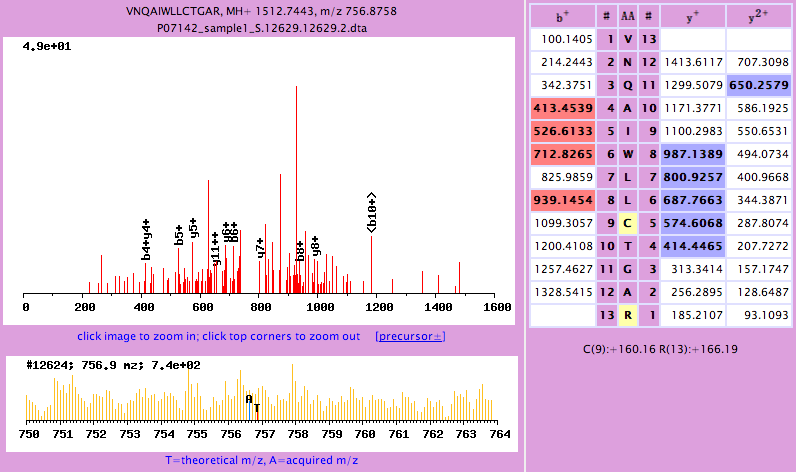


D-dopachrome decarboxylase

STEPCAHLLVSSIGVVGTAEQNR


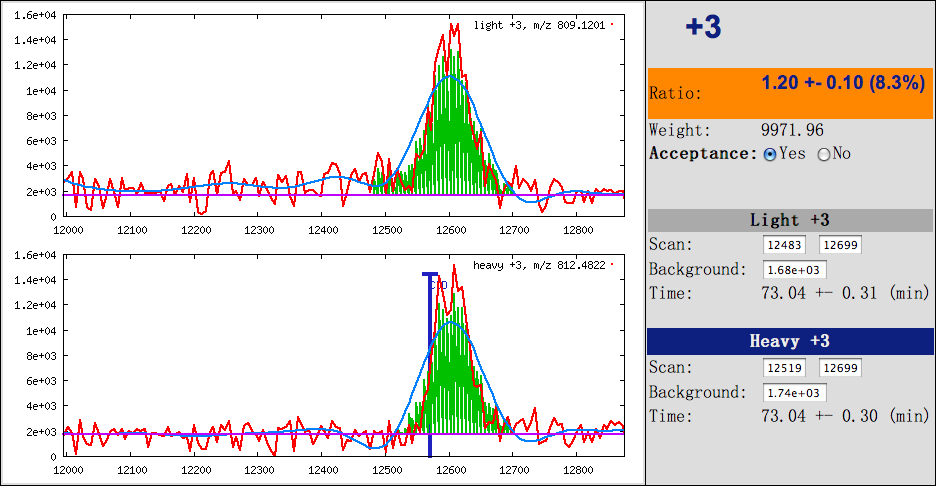

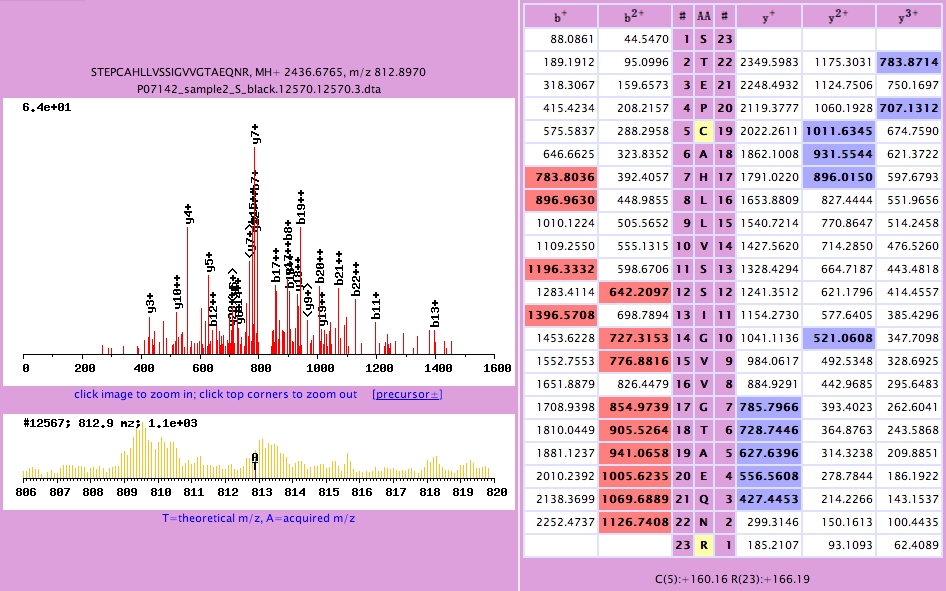


D-3-phosphoglycerate dehydrogenase

NAGTCLSPAVIVGLLR


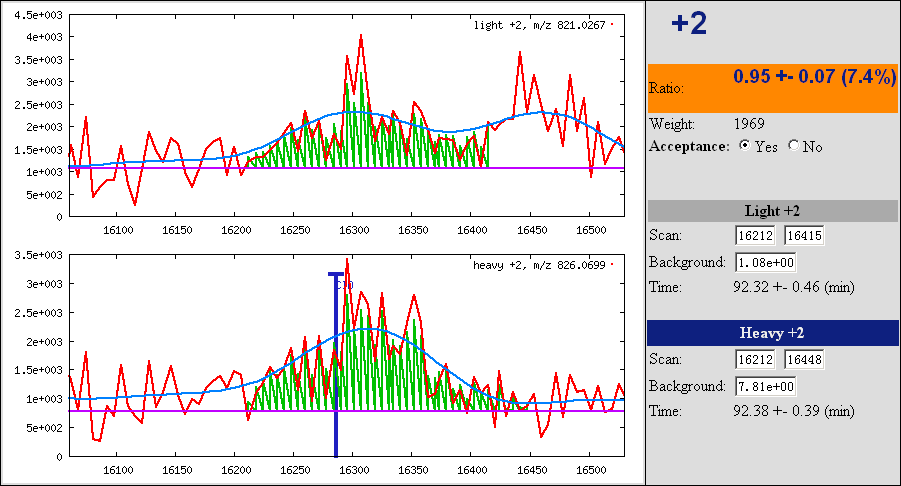

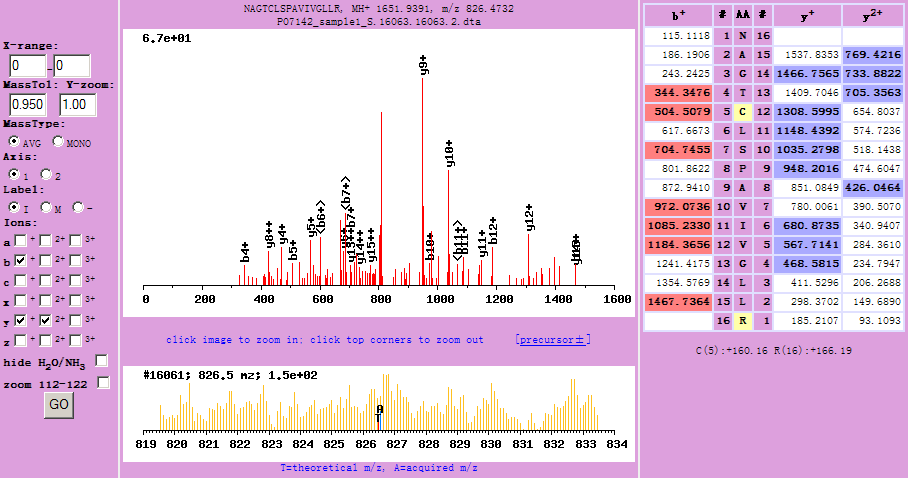


Aspartate aminotransferase

TCGFDFSGALEDISK
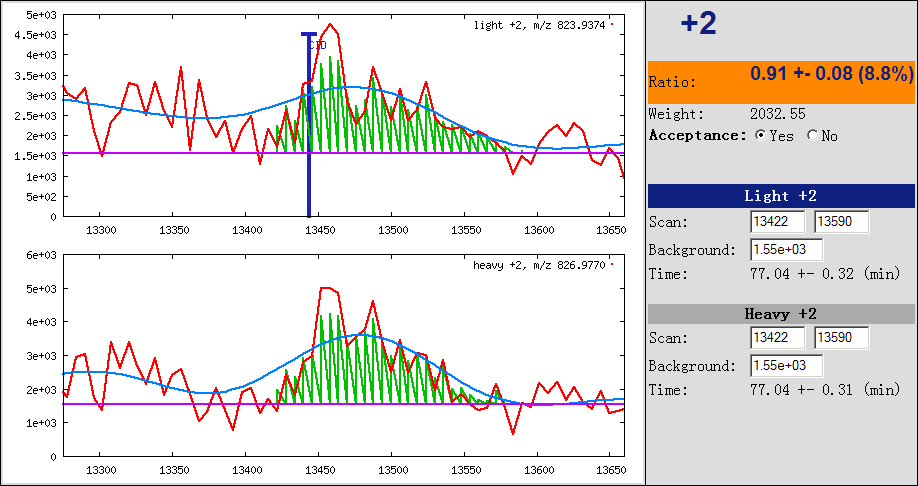

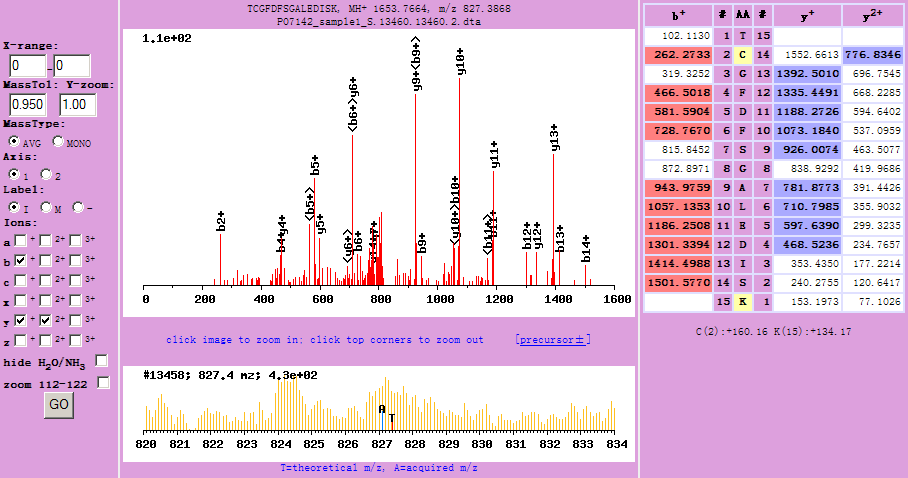


Farnesyl pyrophosphate synthetase

CSWLVVQCLLR
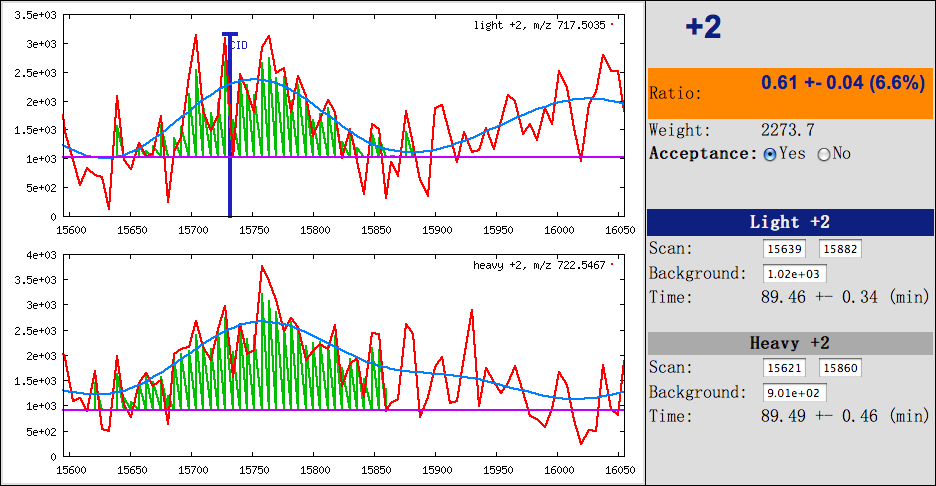

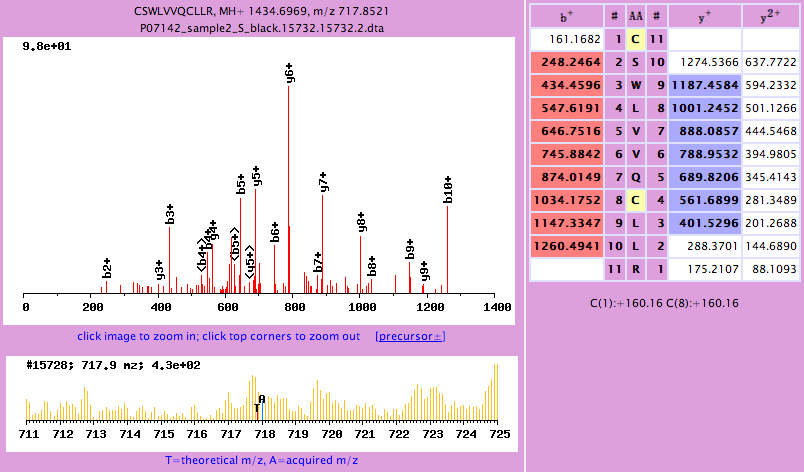


Cathepsin B

EQWSNCPTIGQIR
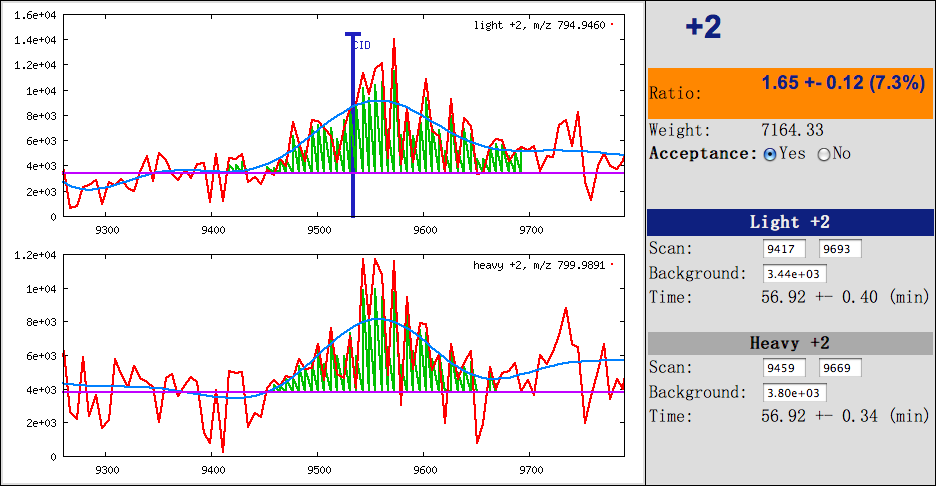

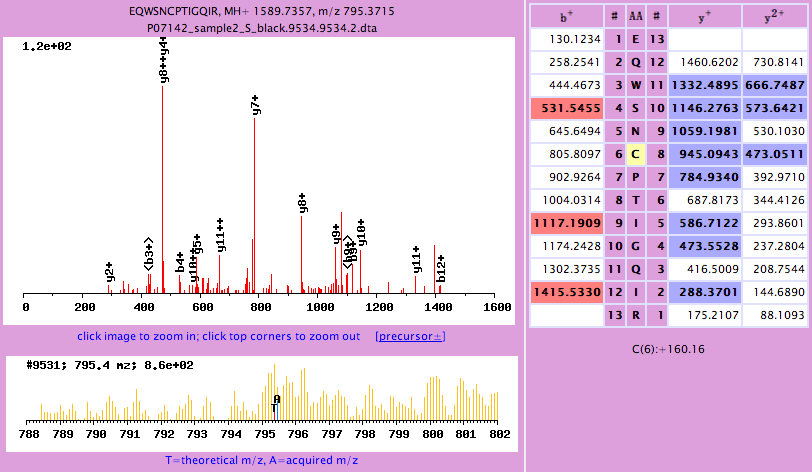


cathepsin S

SGVYDDPSCTGNVNHGVLVVGYGTLDGK


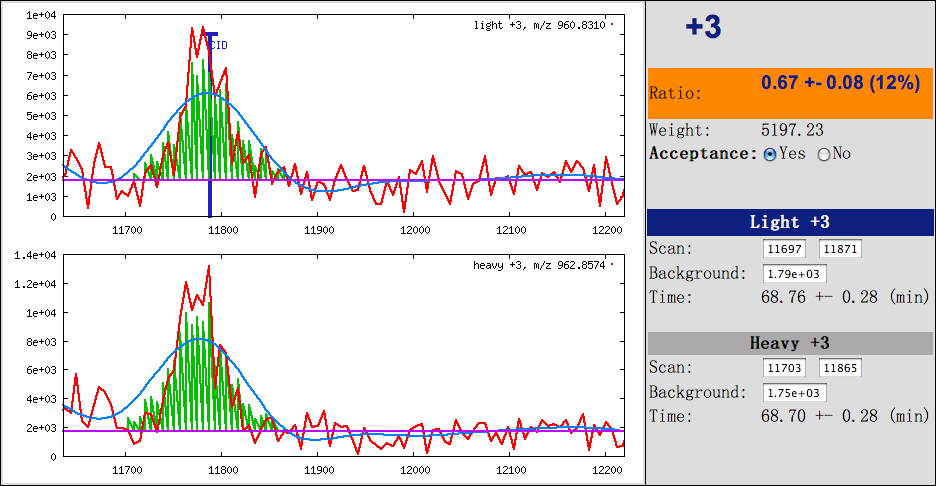

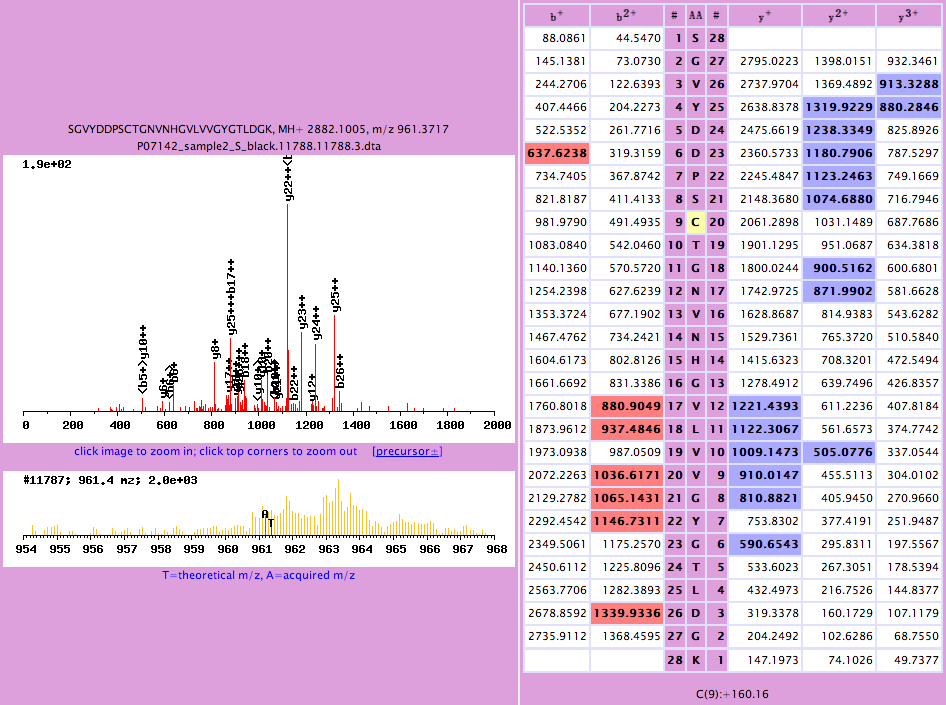


Heat-shock protein 105 kDa

GCALQCAILSPAFK
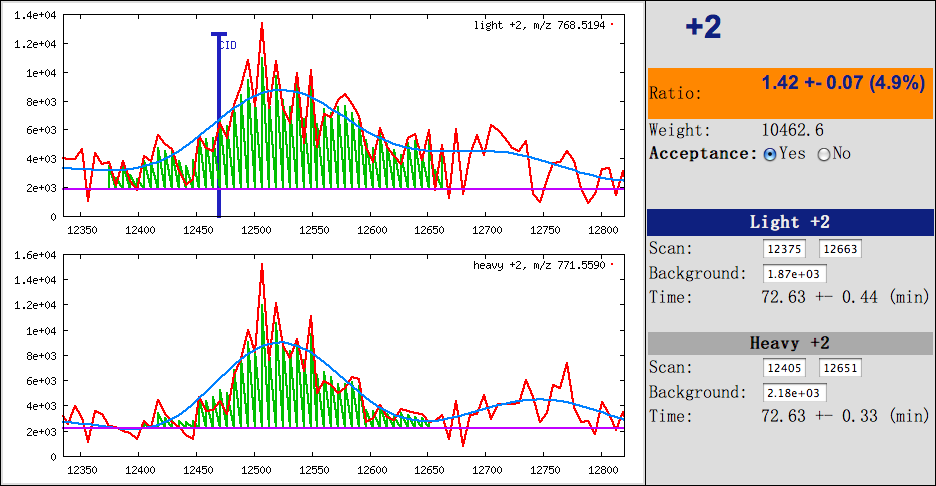

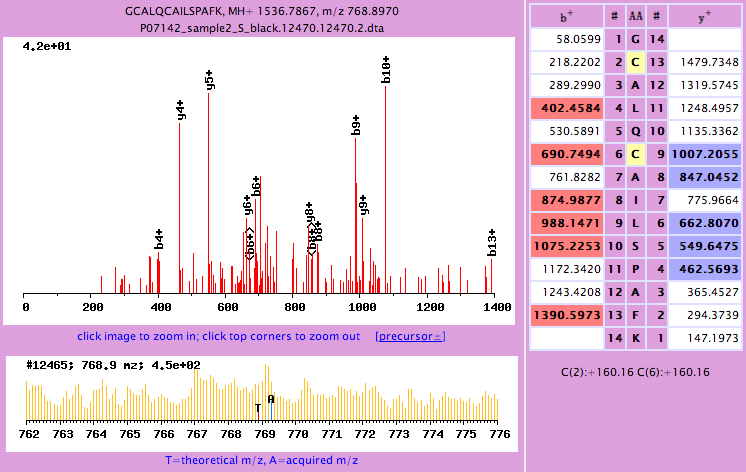


Peroxiredoxin-1

HGEVCPAGWKPGSDTIKPDVNK


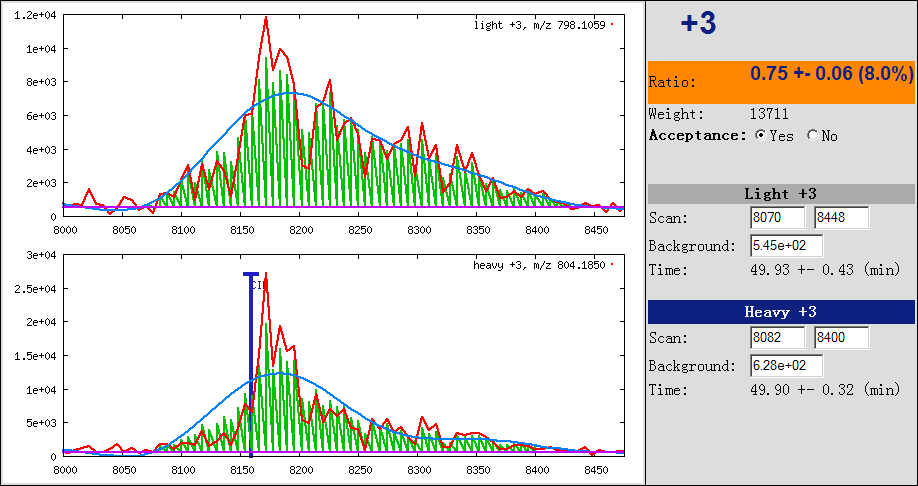

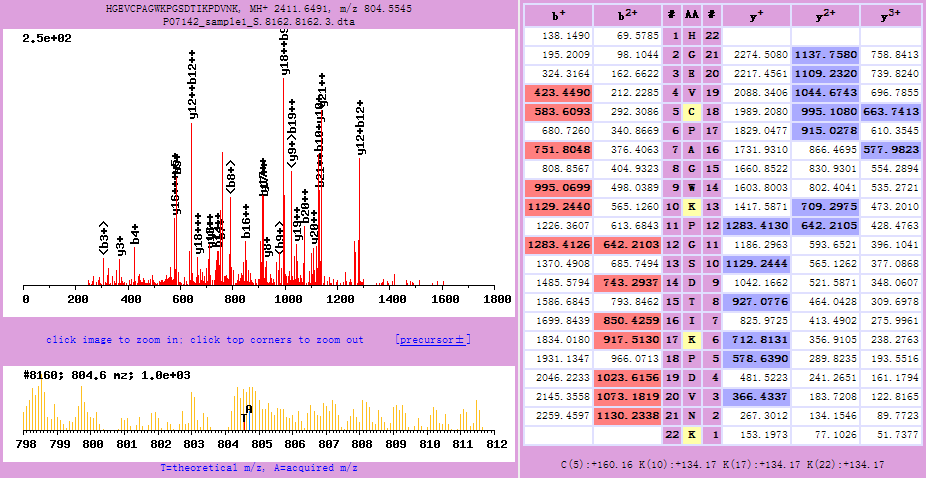


Serpin B6

TCDLLASFK


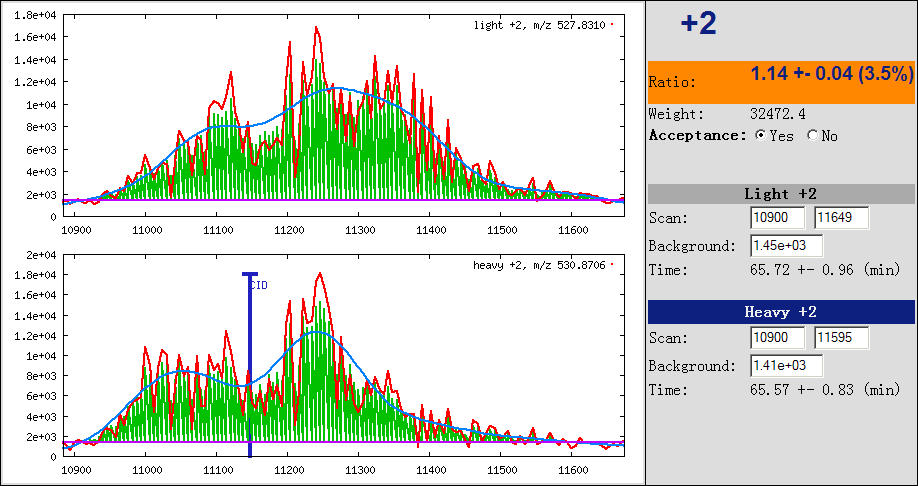

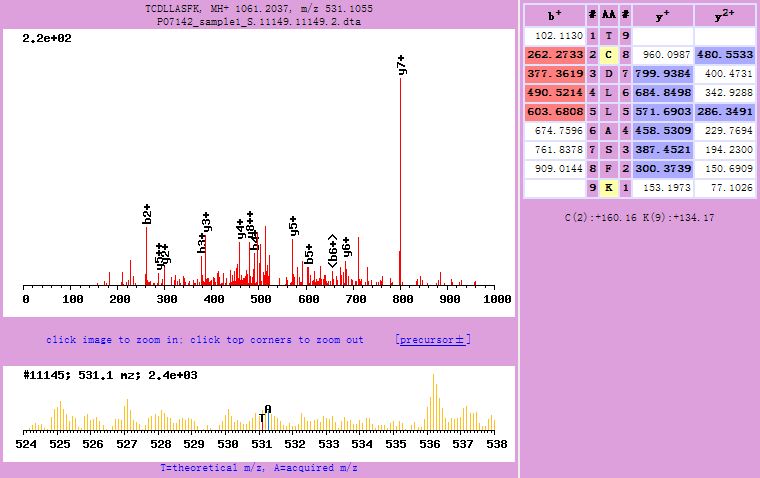


Rho-related GTP-binding protein RhoC

TCLLIVFSK


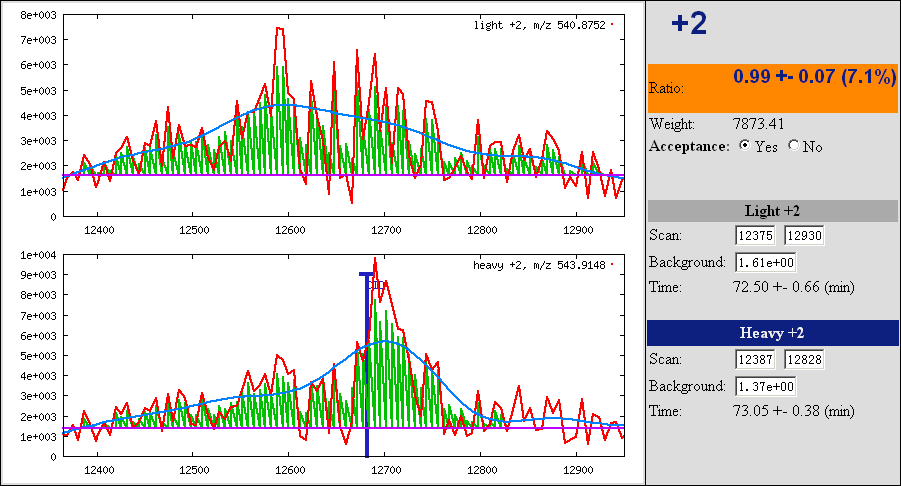


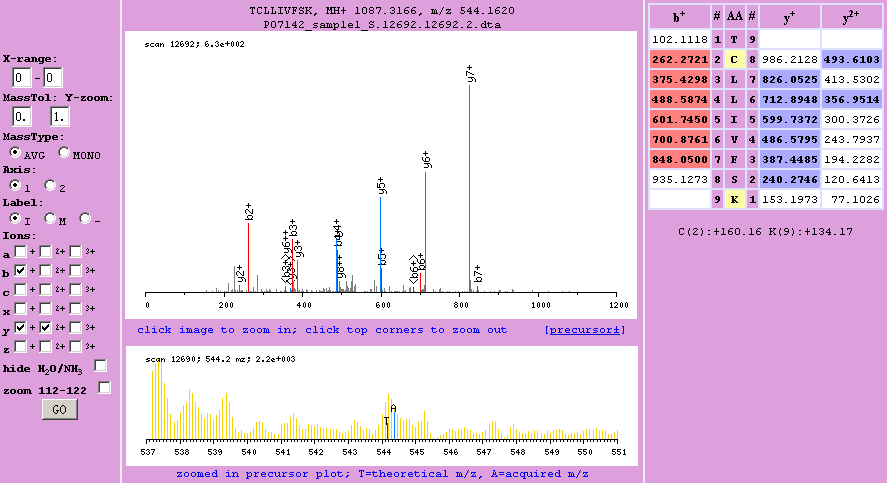


Transketolase

TVPFCSTFAAFFTR


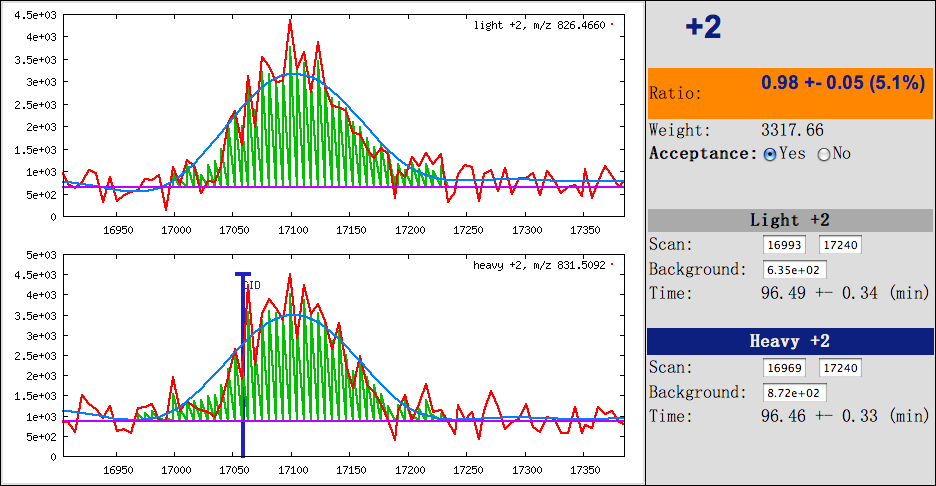

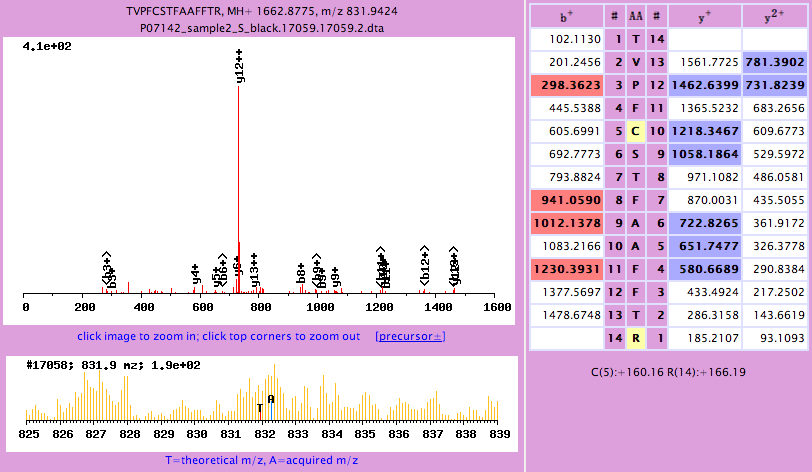


Ubiquitin-activating enzyme E1 X

YFLVGAGAIGCELLK
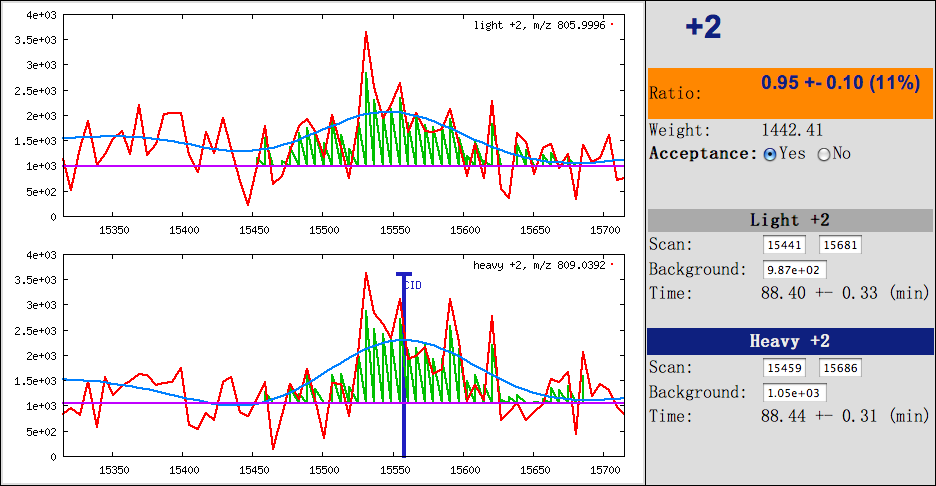

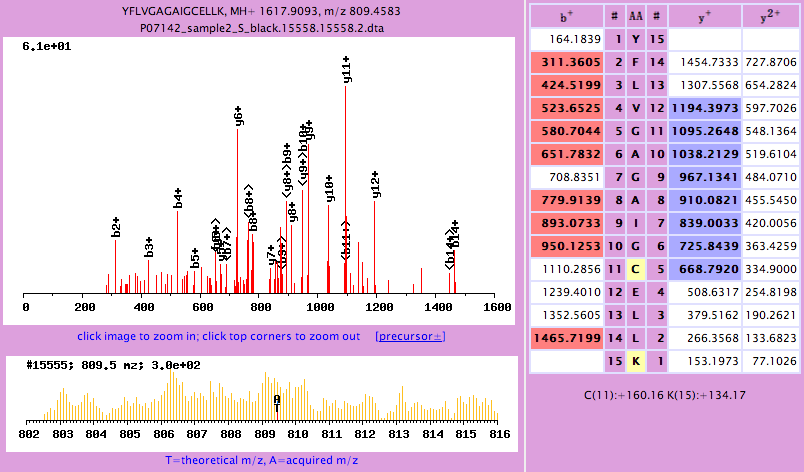


Transaldolase

ALAGCDFLTISPK


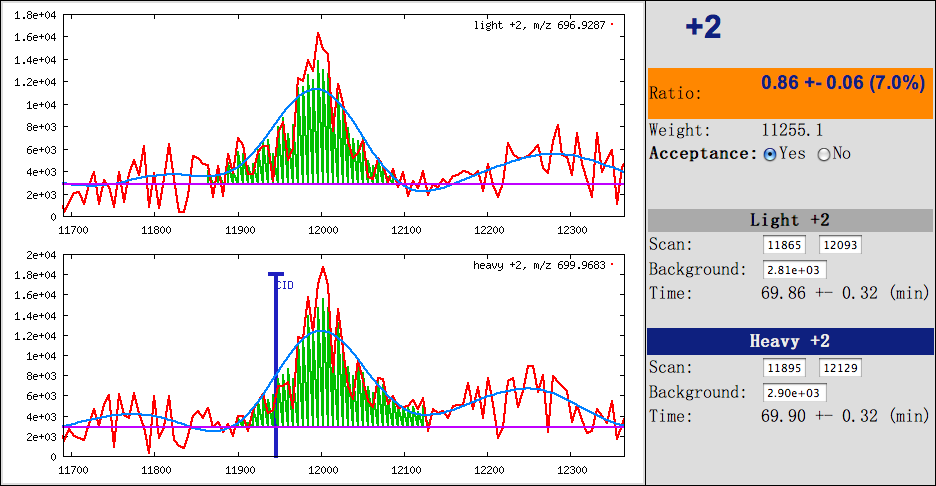

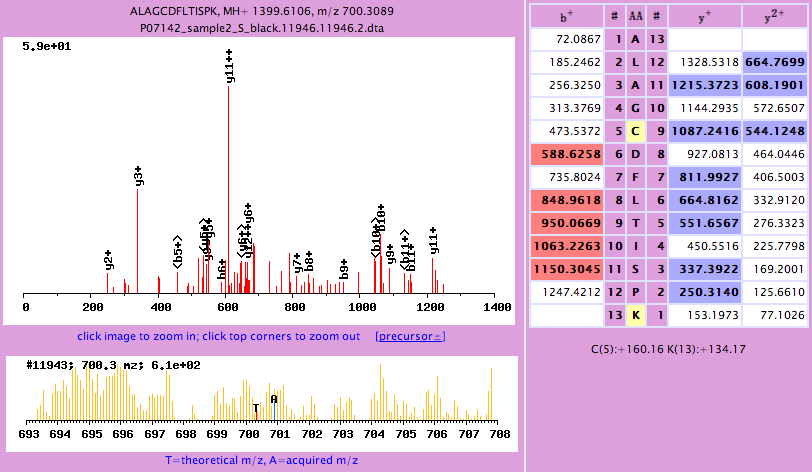


S-formylglutathione hydrolase

CPALYWLSGLTCTEQNFISK
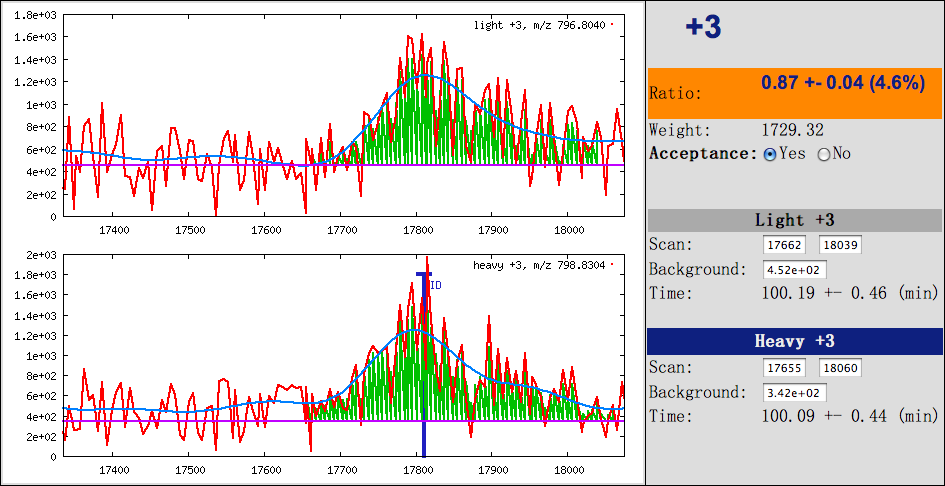

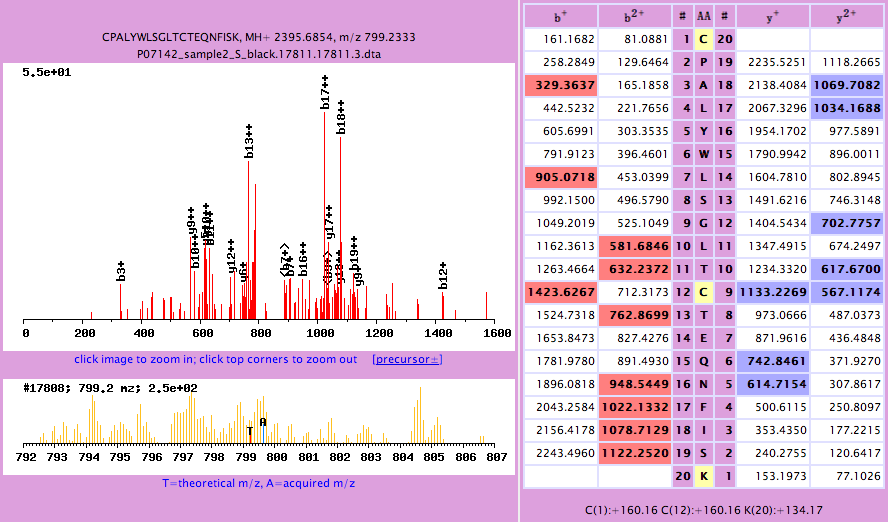


Peroxiredoxin-5

GVLFGVPGAFTPGCSK


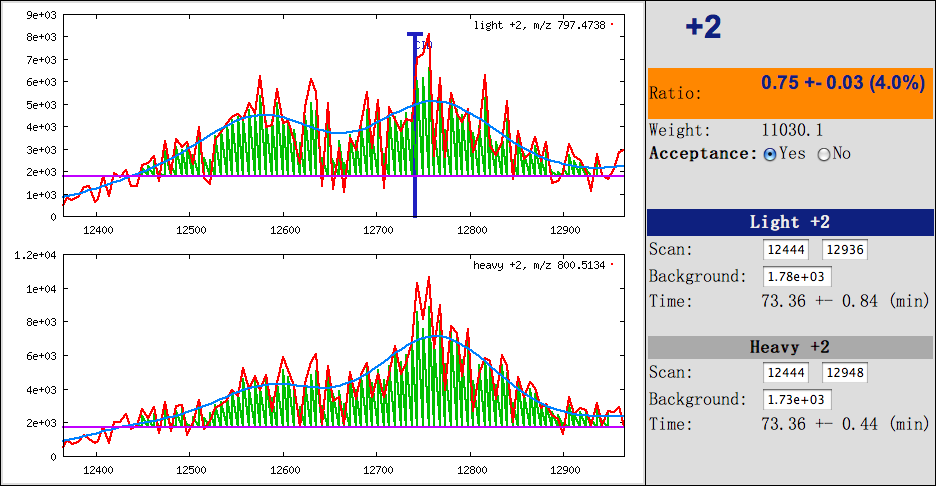

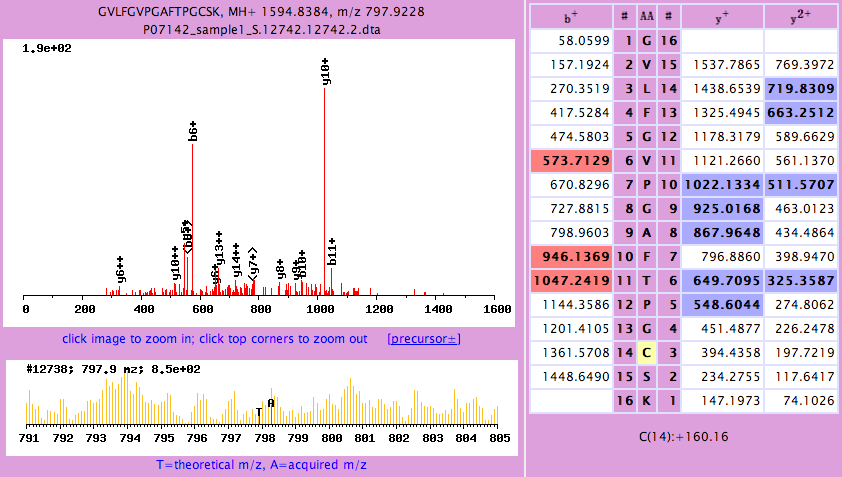


Peroxiredoxin-4

HGEVCPAGWKPGSETIIPDPAGK


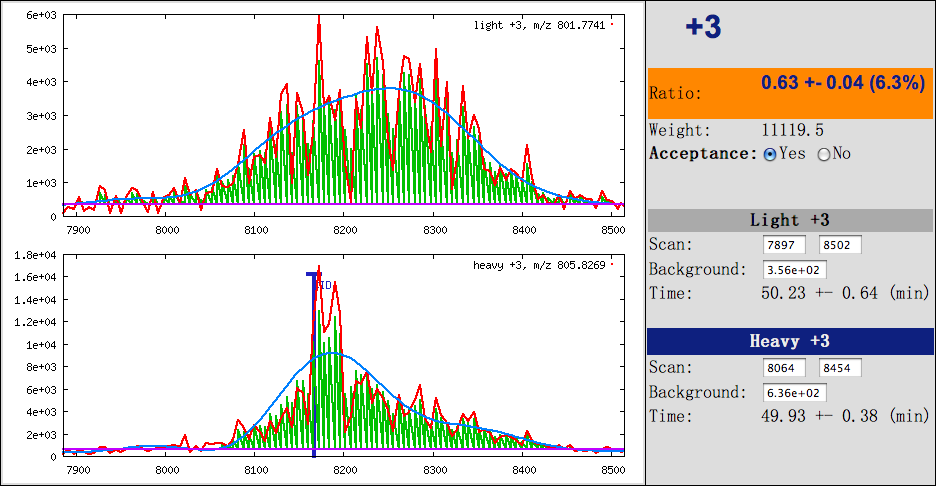

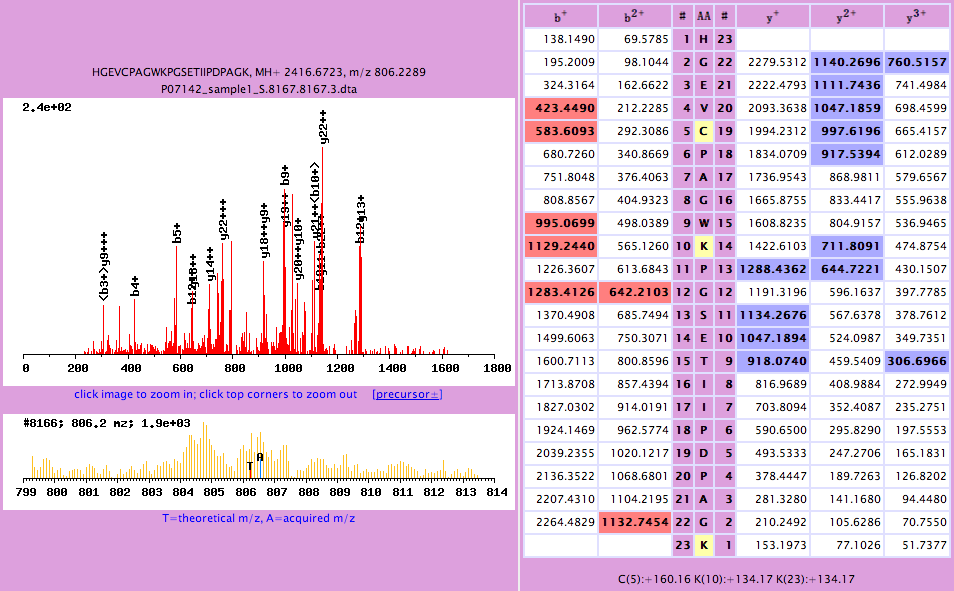


Tubulin alpha-1B chain

SIQFVDWCPTGFK
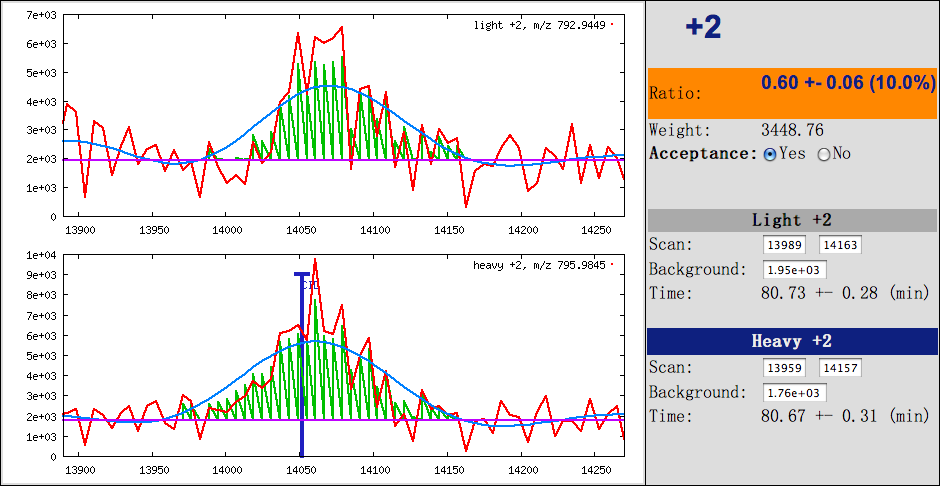

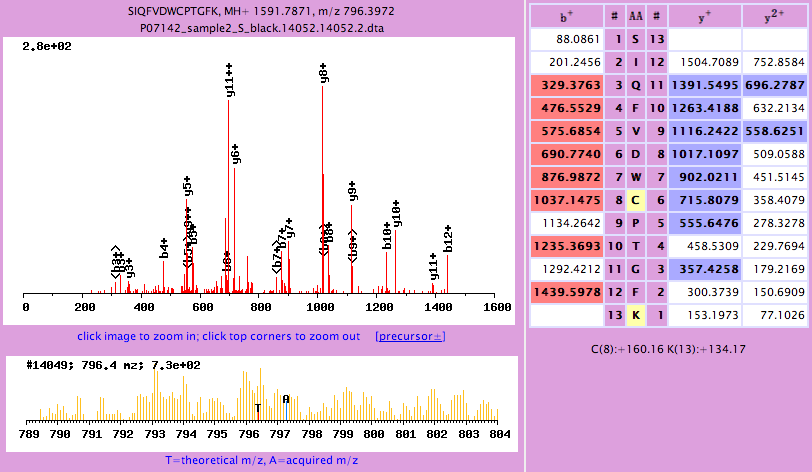

Supplement: Figure S2 — ASAPRatio quantification and MS/MS analysis of all peptides listed in Table 1. (3.17 MB DOC) [file pone.0010015.s002.doc]
