# Supplementary material for: ESNOQ, Proteomic Quantification of Endogenous S-Nitrosation
Source: PLoS One. 2010 Apr 2;5(4):e10015. doi: 10.1371/journal.pone.0010015 (PMC2848867; doi:10.1371/journal.pone.0010015)
Supplement: Table S1 — S-nitrosation quantification with the ESNOQ approach of GSNO treated RAW264.7 cells. RAW264.7 cells labeled with heavy amino acids were treated with 10 µM GSNO for 1 h. Cells labeled with light amino acids were used as control group with the solvent as control treatment. First, cell lysis was blocked with MMTS to avoid the trans-nitrosation, and then the two groups were mixed into one sample. Purification and MS/MS analysis were the same as described in the manuscript. The ratios in this table represent the quantification results of Heavy/Light, i.e. the increasing of S-nitrosation after GSNO treatment. (0.20 MB DOC) [file pone.0010015.s003.doc]

**Table S1.** *S*-nitrosation quantification with the ESNOQ approach of GSNO treated RAW264.7 cells. RAW264.7 cells labeled with heavy amino acids were treated with 10 µM GSNO for 1h. Cells labeled with light amino acids were used as control group with the solvent as control treatment. First, cell lysis was blocked with MMTS to avoid the trans-nitrosation, and then the two groups were mixed into one sample. Purification and MS/MS analysis were the same as described in the manuscript. The ratios in this table represent the quantification results of Heavy/Light, i.e. the increasing of S-nitrosation after GSNO treatment.

| **Swiss-prot** | **Peptide Sequence** | **Ratio** | **SD of Ratio** | **Probability** |
| --- | --- | --- | --- | --- |
| 60S ribosomal protein L4 | | | | |
| Q9D8E6 | FCIWTESAFR | 1.45 | 0.11 | 1 |
| Cathepsin B | | | | |
| P10605 | GENHCGIESEIVAGIPR | 1.11 | 0.09 | 1 |
| P10605 | EQWSNCPTIGQIR | 1.79 | 0.06 | 1 |
| Actin-related protein 3 | | | | |
| Q99JY9 | LPACVVDCGTGYTK | 2.44 | 0.18 | 1 |
|  | YSYVCPDLVK | 1.79 | 0.1 | 0.94 |
| T-complex protein 1 subunit epsilon | | | | |
| P80316 | IAILTCPFEPPKPK | 0.83 | 0.03 | 1 |
| Protein DJ-1 | | | | |
| Q99LX0 | VTVAGLAGKDPVQCSR | 2.27 | 0.21 | 1 |
| 40S ribosomal protein S11 | | | | |
| P62281 | CPFTGNVSIR | 2.56 | 0.39 | 1 |
| Cathepsin Z | | | | |
| Q9WUU7 | HGIPDETCNNYQAK | 1.09 | 0.14 | 0.86 |
| GTP-binding nuclear protein Ran | | | | |
| Q61820 | VCENIPIVLCGNK | 3.03 | 0.18 | 0.99 |
| Pcbp3 protein | | | | |
| Q61990-1 | LVVPASQCGSLIGK | 1.72 | 0.09 | 0.85 |
| ribosomal protein L18A family member | | | | |
| P62717 | DLTTAGAVTQCYR | 2.56 | 1.45 | 0.85 |
| Vimentin | | | | |
| P20152 | QVQSLTCEVDALK | 4.55 | 0.83 | 1 |
| L-lactate dehydrogenase C | | | | |
| P16125 | VIGSGCNLDSAR | 1.75 | 0.06 | 0.99 |
| Galectin-1 | | | | |
| P16045 | DSNNLCLHFNPR | 2.78 | 0.08 | 1 |
| Lamin-B1 | | | | |
| P14733 | CQSLTEDLEFRK | 1.45 | 0.08 | 1 |
| Macrophage migration inhibitory factor isoform 1 | | | | |
| P34884 | LLCGLLSDR | 2.70 | 0.15 | 1 |
| Isoform C2 of Lamin-A\C | | | | |
| P48678-3 | AQNTWGCGSSLR | 1.22 | 0.04 | 1 |
| Ribonuclease inhibitor | | | | |
| Q91VI7 | LLCEGLQDPQCR | 4.00 | 0.48 | 0.93 |
| Malate dehydrogenase | | | | |
| P08249 | GCDVVVIPAGVPR | 2.44 | 0.18 | 1 |
|  | GYLGPEQLPDCLK | 1.52 | 0.11 | 1 |
|  | TIIPLISQCTPK | 2.33 | 0.11 | 1 |
| Inosine 5-phosphate dehydrogenase 2 | | | | |
| P24547 | FVPYLIAGIQHSCQDIGAK | 1.01 | 0.08 | 1 |
| Cofilin-1 | | | | |
| P18760 | AVLFCLSEDKK | 4.17 | 0.35 | 1 |
| Serine hydroxymethyltransferase | | | | |
| Q3TFD0 | AALEALGSCLNNK | 1.64 | 0.05 | 1 |
| triosephosphate isomerase 1 | | | | |
| P17751 | IIYGGSVTGATCK | 1.11 | 0.07 | 1 |
|  | VSHALAEGLGVIACIGEK | 1.23 | 0.09 | 1 |
|  | IAVAAQNCYK | 0.90 | 0.05 | 0.95 |
| Peptidyl-prolyl cis-trans isomerase | | | | |
| P17742 | ITISDCGQL | 2.17 | 0.09 | 1 |
| Phosphoglycerate kinase 1 | | | | |
| P09411 | DCVGPEVENACANPAAGTVILLENLR | 2.38 | 0.23 | 1 |
|  | GCITIIGGGDTATCCAK | 3.03 | 0.09 | 1 |
| Proliferating cell nuclear antigen | | | | |
| P17918 | CAGNEDIITLR | 1.69 | 0.09 | 1 |
| Fatty acid-binding protein | | | | |
| Q05816 | TTVFSCNLGEK | 3.57 | 0.26 | 1 |
| 14-3-3 protein zeta\delta | | | | |
| P63101 | DICNDVLSLLEK | 0.79 | 0.07 | 1 |
| 40S ribosomal protein S12 | | | | |
| P63323 | LGEWVGLCK | 2.94 | 0.17 | 1 |
|  | LVEALCAEHQINLIK | 2.13 | 0.18 | 1 |
|  | VVGCSCVVVK | 1.37 | 0.08 | 0.91 |
| T-complex protein 1 subunit alpha B | | | | |
| P11984 | VLCELADLQDK | 1.25 | 0.16 | 1 |
| Plastin-2 | | | | |
| Q61233 | KLENCNYAVDLGK | 2.86 | 0.49 | 1 |
| Hypothetical protein | | | | |
| P14206 | ADHQPLTEASYVNLPTIALCNTDSPLR | 1.39 | 0.08 | 1 |
| Adenylyl cyclase-associated protein | | | | |
| P40124 | ALLATASQCQQPAGNK | 1.33 | 0.21 | 1 |
| Fructose-bisphosphate aldolase | | | | |
| P05064 | ALANSLACQGK | 2.33 | 0.32 | 1 |
| 60S ribosomal protein L30 | | | | |
| P62889 | LVILANNCPALR | 1.72 | 0.21 | 1 |
| Seryl-aminoacyl-tRNA synthetase | | | | |
| P26638 | TICAILENYQAEK | 1.25 | 0.08 | 1 |
| translation initiation factor eIF-2 gamma subunit | | | | |
| Q9Z0N1 | IVLTNPVCTEVGEK | 1.08 | 0.09 | 1 |
| 60 kDa heat shock protein | | | | |
| P63038-1 | AAVEEGIVLGGGCALLR | 2.00 | 0.12 | 1 |
| ribosomal protein L3 | | | | |
| P27659 | VACIGAWHPAR | 1.85 | 0.14 | 1 |
| Pyruvate kinase isozymes M1\M2 | | | | |
| P52480-1 | NTGIICTIGPASR | 4.00 | 0.16 | 1 |
| P52480-1 | CCSGAIIVLTK | 2.38 | 0.06 | 1 |
| Alpha-enolase | | | | |
| P17182 | SGETEDTFIADLVVGLCTGQIK | 1.35 | 0.13 | 1 |
| P17182 | VNQIGSVTESLQACK | 2.38 | 0.06 | 0.98 |
| Elongation factor 2 | | | | |
| P58252 | ETVSEESNVLCLSK | 2.22 | 0.1 | 1 |
|  | STLTDSLVCK | 1.41 | 0.08 | 1 |
|  | CLYASVLTAQPR | 1.18 | 0.07 | 0.89 |
| ribosomal protein S8 | | | | |
| P62242 | NCIVLIDSTPYR | 2.08 | 0.26 | 1 |
| Transitional endoplasmic reticulum ATPase | | | | |
| Q01853 | AIANECQANFISIK | 1.96 | 0.15 | 1 |
| 60S ribosomal protein L12 | | | | |
| P35979 | CTGGEVGATSALAPK | 2.22 | 0.15 | 1 |
| Heterogeneous nuclear ribonucleoprotein D0 | | | | |
| Q60668-2 | FGEVVDCTLK | 4.35 | 0.19 | 0.98 |
| Ribosomal protein S17 | | | | |
| P63276 | VCEEIAIIPSKK | 4.17 | 0.69 | 1 |
| Spliceosome RNA helicase Bat1 | | | | |
| Q9Z1N5 | NCPHIVVGTPGR | 1.27 | 0.05 | 0.99 |
| RAN GTPase activating protein 1 | | | | |
| P46061 | SPACFTLQELK | 1.64 | 0.08 | 0.99 |
| High mobility group protein B2 | | | | |
| P30681 | RPPSAFFLFCSENRPK | 1.16 | 0.07 | 1 |
| Actin-related protein 2\3 complex subunit 2 | | | | |
| Q9CVB6 | NCFASVFEK | 1.09 | 0.05 | 0.69 |
| Ubiquitin-like modifier-activating enzyme 1 | | | | |
| Q02053 | DNPGVVTCLDEAR | 1.32 | 0.05 | 0.99 |
| 26S proteasome non-ATPase regulatory subunit 2 | | | | |
| Q8VDM4 | SGALLACGIVNSGVR | 1.09 | 0.07 | 1 |
| Putative uncharacterized protein | | | | |
| O35737 | GLPWSCSADEVQR | 1.02 | 0.05 | 1 |
| 60S ribosomal protein | | | | |
| P12970 | TCTTVAFTQVNSEDKGALAK | 1.64 | 0.11 | 1 |
| Acidic leucine-rich nuclear phosphoprotein 32 | | | | |
| O35381 | SLDLFNCEVTNLNAYR | 3.85 | 0.3 | 1 |
| Phosphoglycerate mutase 1 | | | | |
| Q9DBJ1 | YADLTEDQLPSCESLKDTIAR | 2.38 | 0.11 | 1 |
| ribosomal protein S27a | | | | |
| P62983 | CCLTYCFNKPEDK | 0.81 | 0.09 | 1 |
| 60S ribosomal protein L23 | | | | |
| P62830 | ISLGLPVGAVINCADNTGAK | 1.79 | 0.1 | 1 |
| Transaldolase | | | | |
| Q93092 | ALAGCDFLTISPK | 1.27 | 0.06 | 1 |
| S-formylglutathione hydrolase | | | | |
| Q9R0P3 | SVSAFAPICNPVLCSWGK | 1.85 | 0.21 | 1 |
| Isoform 2 of Muscleblind-like protein 2 | | | | |
| Q8C181-2 | VIACFDSLK | 1.52 | 0.09 | 1 |
| Malate dehydrogenase 1 | | | | |
| P14152, | VIVVGNPANTNCLTASK | 1.56 | 0.12 | 1 |
| 40S ribosomal protein S21 | | | | |
| Q9CQR2 | TYGICGAIR | 1.05 | 0.01 | 1 |
| Filamin-B | | | | |
| Q80X90 | CLATGPGIAPTVK | 2.00 | 0.16 | 1 |
| Ribosomal protein L10A | | | | |
| Q3U561 | FSVCVLGDQQHCDEAK | 2.00 | 0.08 | 1 |
| Glycosyltransferase 25 family member 1 | | | | |
| Q8K297 | ALHEQEIDCQLVEAVDGK | 1.11 | 0.09 | 1 |
| Proteasome subunit alpha type-6 | | | | |
| Q9QUM9 | CDPAGYYCGFK | 0.94 | 0.05 | 1 |
| Regulator of differentiation 1 | | | | |
| Q8BHD7-1 | NLFTEAGCSVK | 1.09 | 0.07 | 1 |
| SWI\SNF complex subunit SMARCC1 | | | | |
| P97496-1 | AGGTLCHILGAAYK | 1.09 | 0.04 | 1 |
| Heterogeneous nuclear ribonucleoprotein A3 | | | | |
| Q8BG05-1 | YHTINGHNCEVKK | 1.23 | 0.05 | 1 |
| Eukaryotic translation initiation factor 6 | | | | |
| O55135 | ASFENNCEVGCFAK | 1.54 | 0.12 | 0.99 |
| Gcsh protein | | | | |
| Q91WK5 | SCYEDGWLIK | 2.56 | 0.07 | 0.99 |
| Ribosomal protein S6 | | | | |
| P62754 | LNISFPATGCQK | 1.35 | 0.05 | 0.99 |
| Hsp90 co-chaperone Cdc37 | | | | |
| Q61081 | CIDSGLWVPNSK | 1.05 | 0.06 | 0.99 |
| ATP-dependent RNA helicase DDX17 | | | | |
| Q501J6-1 | GDGPICLVLAPTR | 1.22 | 0.07 | 0.98 |
| Proliferation-associated protein 2G4 | | | | |
| P50580 | AAHLCAEAALR | 1.52 | 0.07 | 0.97 |
| DNA replication licensing factor MCM4 | | | | |
| P49717 | AGIICQLNAR | 20.35 | 4.17 | 0.97 |
| ribosomal protein L36 | | | | |
| P47964 | EVCGFAPYER | 8.33 | 0.69 | 0.97 |
| T-complex protein 1 subunit beta | | | | |
| P80314 | HGINCFINR | 1.25 | 0.05 | 0.97 |
| Glucosamine--fructose-6-phosphate aminotransferase | | | | |
| P47856-1 | CQNALQQVVAR | 2.08 | 0.17 | 0.95 |
